# Two-fold addition reaction of silylene to C_60_: structural and electronic properties of a bis-adduct

**DOI:** 10.3762/bjoc.20.100

**Published:** 2024-05-22

**Authors:** Masahiro Kako, Masato Kai, Masanori Yasui, Michio Yamada, Yutaka Maeda, Takeshi Akasaka

**Affiliations:** 1 Department of Engineering Science, The University of Electro-Communications, Chofu 182-8585, Japan; 2 Department of Chemistry, Tokyo Gakugei University, Tokyo 184-8501, Japan; 3 TARA Center, University of Tsukuba, Tsukuba 305-8577, Japan

**Keywords:** bis-adduct, C_60_, fullerene, silirane, silylene

## Abstract

The addition reaction of C_60_ with silylene **1**, a silicon analog of carbene, yielded the corresponding bis-adduct **3**. The structure of **3** was determined by single-crystal X-ray structure analysis, representing the first example of a crystal structure of a silirane (silacyclopropane) derivative of fullerenes. Electrochemical measurements confirmed that the redox potentials of **3** are shifted cathodically compared to those of the parent mono-adduct **2**. Density functional theory (DFT) calculations provided the basis for the electronic properties of compound **3**.

## Introduction

The chemical functionalization of fullerenes has been exploited extensively from both fundamental and practical perspectives, elucidating their potential applications for biochemistry, nanomaterials sciences, and molecular electronics [1–3]. With the development of research investigating the functionalization of fullerenes, several multiple addition reactions of fullerenes have been investigated [4–8]. For example, earlier reports have described that some functionalization reactions of fullerenes afford bis-adducts with excellent properties as organic photovoltaic materials [9–14]. Furthermore, regioisomerically pure bis-functionalized fullerenes function better as electron acceptors in organic thin-film solar cells than mixtures of the corresponding regioisomers do [12–14]. Therefore, controlling the regioselectivities of multiple functionalizations of fullerenes for the selective synthesis of specific multi-adducts poses an important challenge.

**Figure 1:**
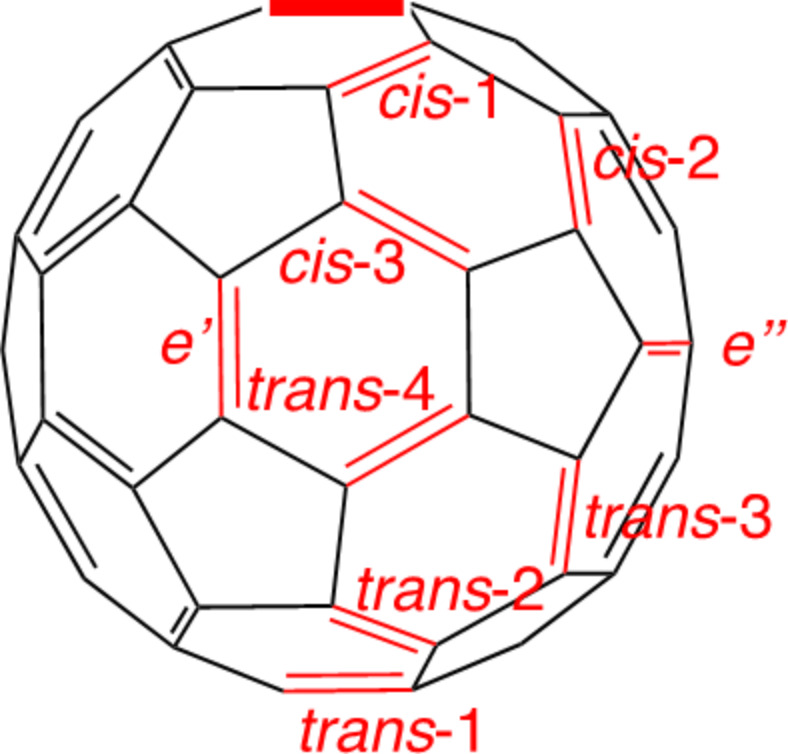
Positional notation of 6,6-bonds in a mono-adduct of C_60_ with the first addition site indicated using a bold line [5].

Addition reactions of C_60_ are well known to occur mainly at the 6,6-bonds (junction between two hexagons) rather than at the 5,6-bonds (junction between a pentagon and a hexagon). If the second additions proceed also at the 6,6-bonds, then nine regioisomers are possible for bis-additions, as shown in Figure 1 [4–5]. In addition, when the second addends are identical to the first, the *e'* and *e''* isomers are the same adducts, thereby affording eight possible regioisomers for bis-addition reactions. Hirsch and co-workers reported two-fold additions of C_60_ using the Bingel–Hirsch and the Bamford–Stevens reactions as well as nitrene addition reactions, indicating the formation of regioisomers as anticipated from the possible addition sites in Figure 1 [5]. Diederich and co-workers developed a general methodology using tether-directed remote functionalization for the regioselective formation of multiple adducts of fullerenes [6].

**Scheme 1:**
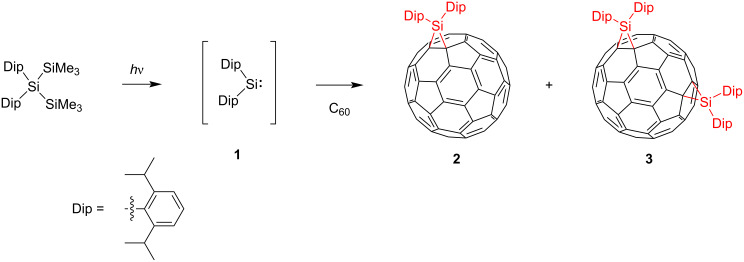
Synthesis of silylene adducts **2** and **3**.

In our earlier reports, the reactions of C_60_ and C_70_, with silylene Dip_2_Si (**1**, Dip = 2,6-diisopropylphenyl), a silicon analog of carbene, were described as providing silirane (silacyclopropane)-type 6,6-mono-adducts Dip_2_SiC_60_ (**2**, Scheme 1) and Dip_2_SiC_70_ [15–16]. Furthermore, bis-adducts (Dip_2_Si)_2_C_60_ and (Dip_2_Si)_2_C_70_ were obtained as byproducts, but no details of these bis-adducts have been clarified [15–16]. The results demonstrated that the electronic properties of product **2** were altered considerably compared to that of pristine C_60_ mainly because silicon atoms are less electronegative. Electron-donating effects of silyl groups in fullerene derivatives were also rationalized in terms of σ–π conjugations between C–Si σ bonds of the silirane ring and adjacent π-bonds of the fullerene cage [17–20]. Therefore, it is interesting to examine the electronic properties of multi-silylene adducts in comparison with those of **2**. Herein, we report the structural determination and characterization of a bis-silylene adduct **3** of C_60_ based on spectroscopic measurements, X-ray crystallography, electrochemical analyses, and theoretical calculations.

## Results and Discussion

### Synthesis of bis-silylene adduct **3**

The synthesis of the silylene adduct was conducted using a modified literature procedure [15]. A degassed solution of Dip_2_Si(SiMe_3_) as a silylene precursor and C_60_ in toluene was irradiated by a 125-W low-pressure mercury lamp in a quartz tube for 2 h (Scheme 1). During reaction, the color of the solution turned to dark brown. After the photolysis, the bis-silylene adduct **3** was isolated in 36% yield accompanied by mono-adduct **2** (22% yield) by silica gel flash column chromatography using mixed solvents of hexane/CH_2_Cl_2_ by changing the ratios of volumes from 10:1 to 3:1. In thin-layer chromatography (TLC) analysis using silica gel, the *R*_f_ values are 0.55 for **2** and 0.33 for **3**, respectively, with hexane/CH_2_Cl_2_ 5:1 as solvent mixture. Although several other fractions containing C_60_ derivatives were obtained, their structures are still under investigation because of the difficulty in isolating and purifying those fractions.

**Figure 2:**
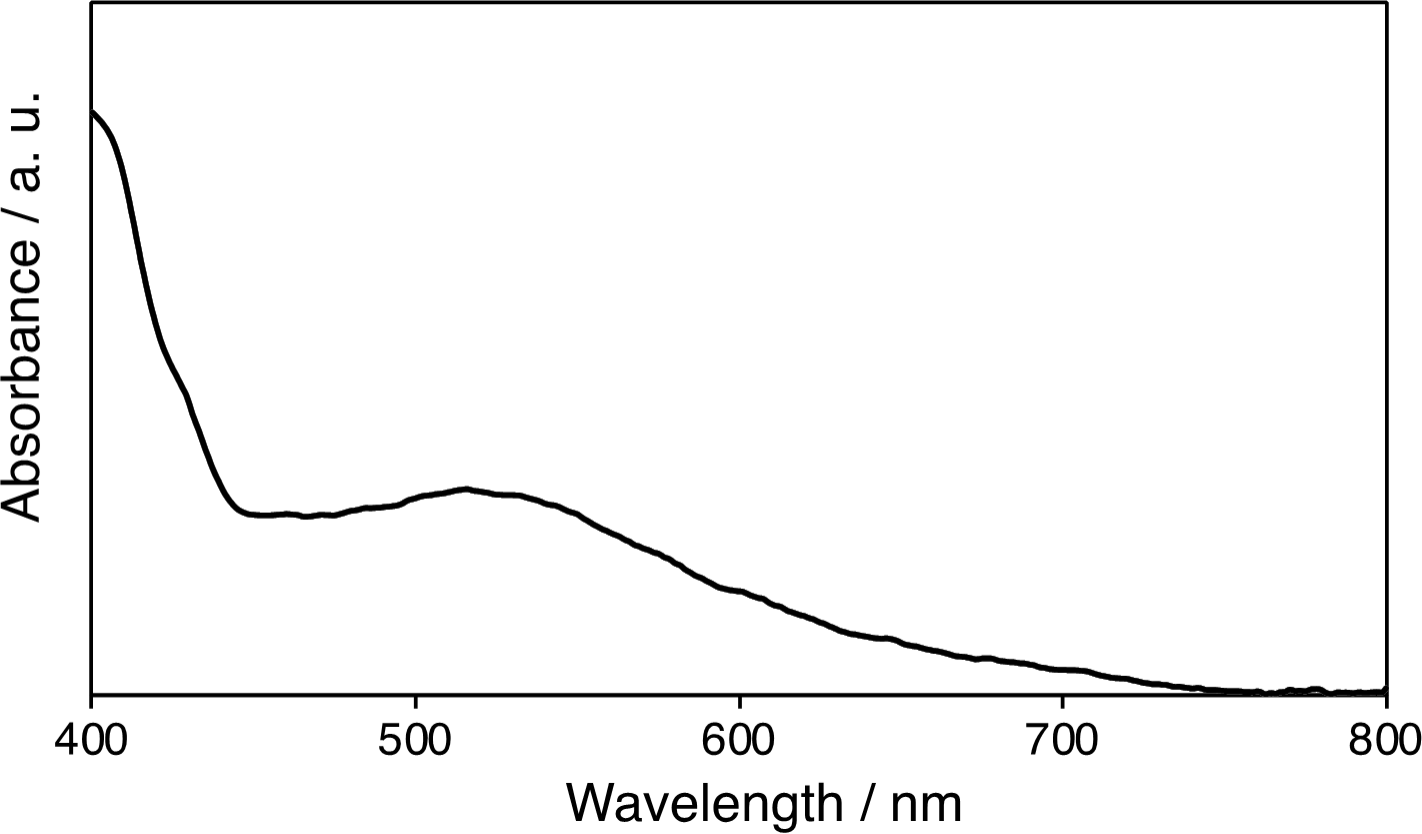
Absorption spectrum of **3** in CH_2_Cl_2_.

To clarify the addition site of **3**, we measured the ultraviolet–visible (UV–vis) spectrum. Fullerene derivatives are well-known to show characteristic absorption spectra depending on the addition pattern. As shown in Figure 2, the spectrum of **3** exhibited an absorption maximum at 515 nm, which is similar to those of the *e′* and *e′′* isomers of C_60_[C(C_6_H_4_OMe)_2_]_2_ and C_60_[C(C_6_H_4_OMe)_2_][NCOOEt] [5]. In the case of **3**, the *e′* and *e′′* isomers are identical products, and **3** is denoted as the *e* isomer hereinafter.

**Figure 3:**
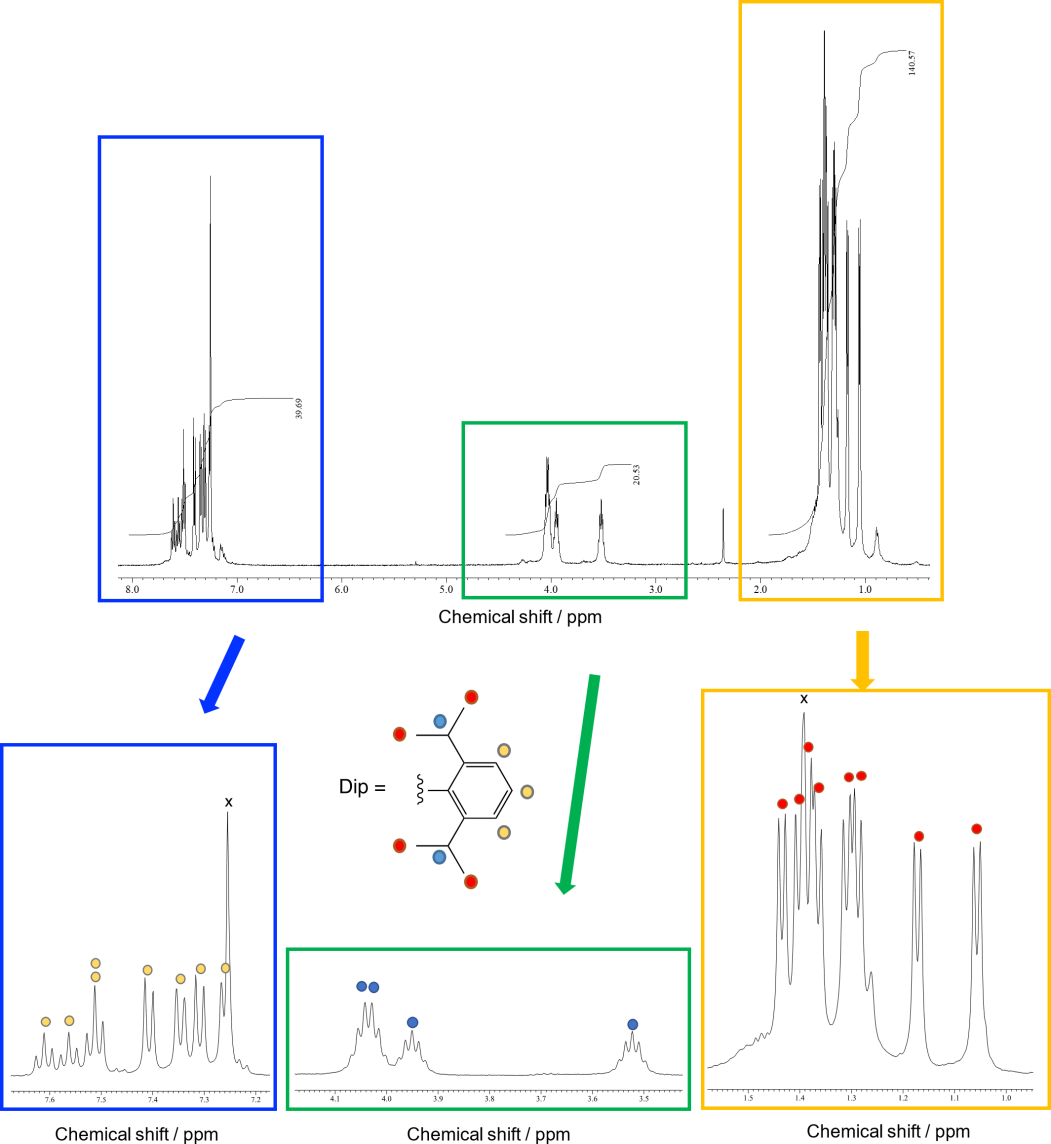
500 MHz ^1^H NMR spectrum of **3** in CDCl_3_/CS_2_ 3:1.

**Figure 4:**
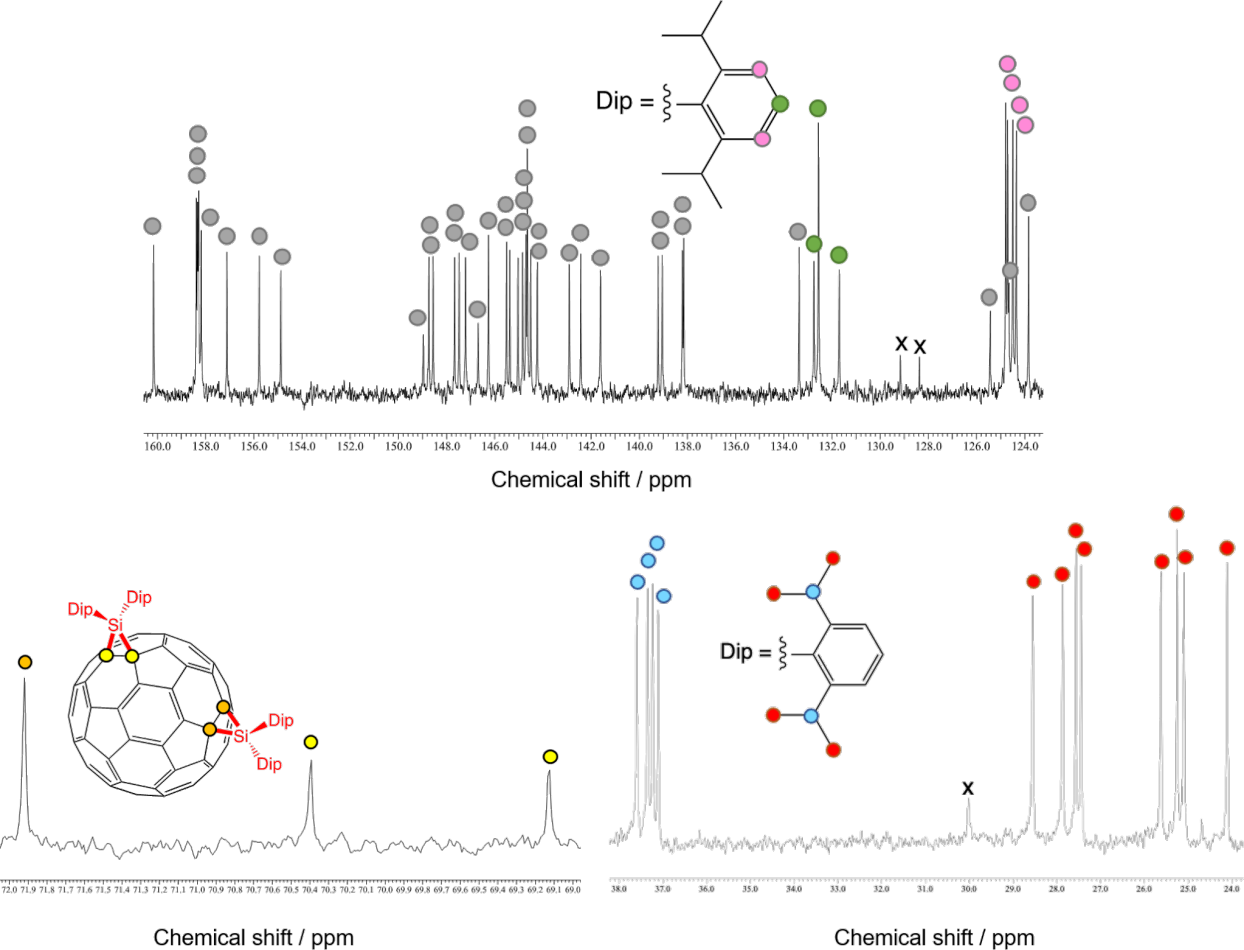
125 MHz ^13^C NMR spectrum of **3** in CDCl_3_/CS_2_ 3:1. The signals of sp^2^ carbons of C_60_ and quaternary carbons of the Dip groups are indicated by grey circles.

The ^1^H NMR spectrum of **3** showed signals of eight doublets and three septets, which were assigned respectively to the methyl and methine groups, along with the aromatic protons (Figure 3). In the ^13^C NMR spectrum, a total of 43 signals were observed in the low-field region (approximately 160–120 ppm), of which 29 signals are attributed to the C_60_ carbon cage and 14 signals are attributed to the aromatic ring carbon nuclei of the Dip groups (Figure 4). In addition, three sp^3^ carbon atoms of the C_60_ carbon cage, eight methyl, and three methine carbon signals of the Dip group were observed. These spectral features are consistent with the structure of **3** as the *e* isomer of bis-adducts with *C*_s_ symmetry. The plane of symmetry includes one silirane ring and bisects another silirane ring perpendicularly.

Unfortunately, the matrix-assisted laser desorption ionization time-of-flight (MALDI-TOF) mass spectrometry of **3** afforded no molecular ion peak expected for adducts derived from Dip_2_Si and C_60_ while a base peak at *m*/*z* 720 due to C_60_ was observed probably because of the low stability of radical ions of **3**.

**Figure 5:**
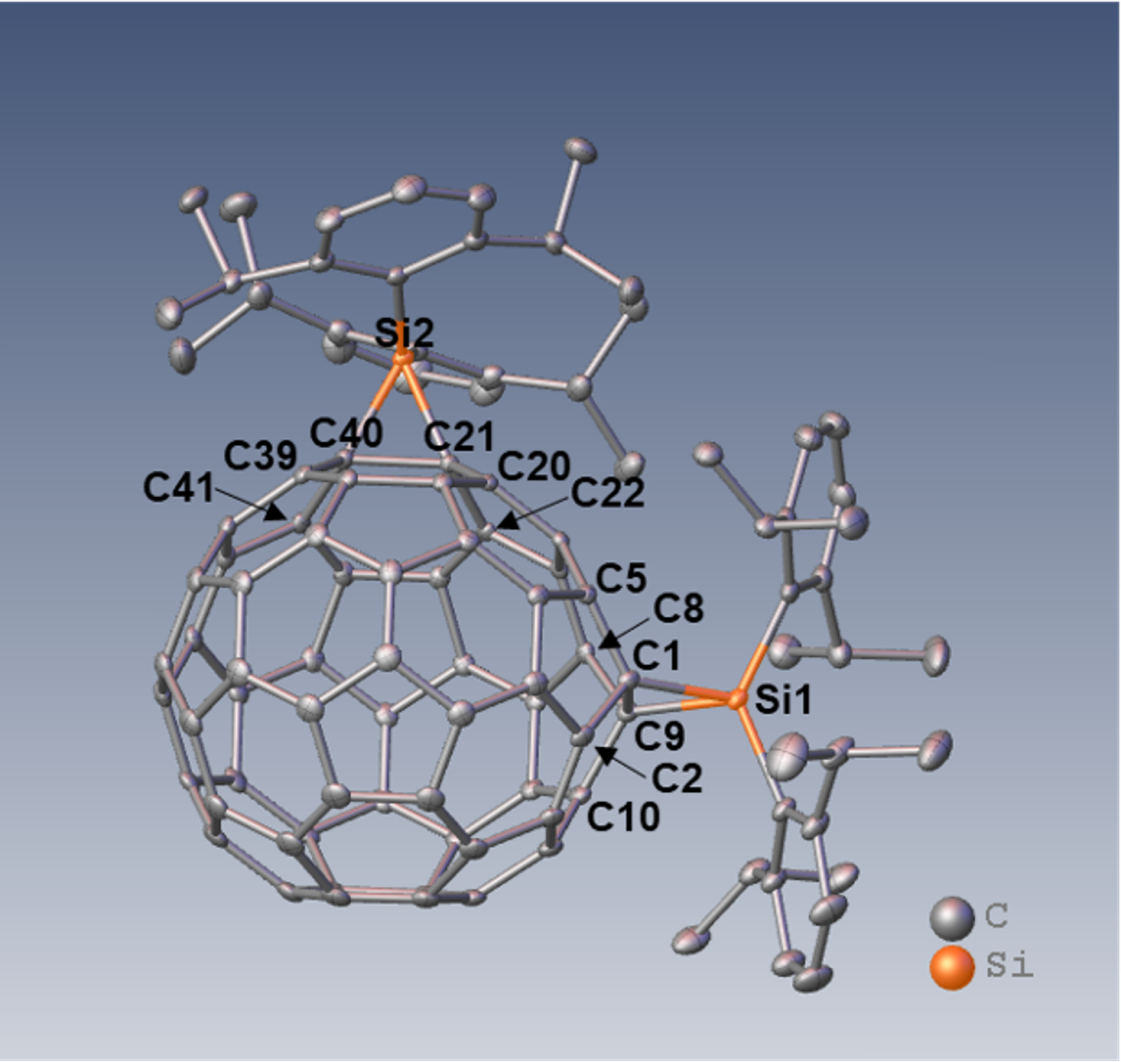
ORTEP drawing of **3** showing thermal ellipsoids at the 50% probability level at 100 K. Hydrogen atoms are omitted for clarity.

Finally, the structure of **3** was established by single-crystal X-ray structure analysis. The ORTEP diagram of **3** is presented in Figure 5 with the selected bond lengths and angles collected in Table 1. The cage C–C bond lengths of the addition sites are C1–C9: 1.623(2) Å and C21–C40: 1.6282(19) Å, which fall within the range of the corresponding values reported for the crystal structures of methano-derivatives of C_60_ [5,21–33]. It is noteworthy that these C–C bond lengths are longer than those of the reported siliranes [34–42], except for rare examples such as 3-(hydroxydimesitylsilyl)-1,1-dimesityl-2,2-bis(trimethylsilyl)-1-silirane [43]. In contrast, we earlier reported the crystal structure of the adduct of Lu_3_N@*I*_h_–C_80_ with silylene Dep_2_Si (Dep = 2,6-diethylphenyl), which was revealed to be a sila-fulleroid structure with the cage C–C separation distance of 2.25 Å at the addition site [44]. On the other hand, the Si–C bond lengths of the silirane ring in **3** are Si1–C1: 1.8886(15) Å, Si1–C9: 1.8796(15) Å, Si2–C21: 1.8778(14) Å, and Si2–C40: 1.8865(15) Å, which are roughly equal to those of the reported siliranes. These results confirm that the two addends in **3** are both silirane structures, which represents the first example of a crystallographic analysis of silirane derivatives of fullerenes.

**Table 1:** Selected bond lengths and angles of **3**.

bond length [Å]		bond angle [°]	

C1–C9	1.623(2)	Si1–C1–C9	64.19(7)
Si1–C1	1.8886(15)	C1–Si1–C9	51.04(6)
Si1–C9	1.8796(15)	Si1–C9–C1	64.77(7)
C1–C2	1.495(2)		
C1–C5	1.497(2)		
C8–C9	1.492(2)		
C9–C10	1.498(2)		
C21–C40	1.6282(19)	Si2–C21–C40	64.65(7)
Si2–C21	1.8778(14)	C21–Si2–C40	51.26(6)
Si2–C40	1.8865(15)	Si2–C40–C21	64.10(7)
C20–C21	1.4951(19)		
C21–C22	1.490(2)		
C39–C40	1.4951(19)		
C40–C41	1.4928(19)		

### Theoretical calculations

**Figure 6:**
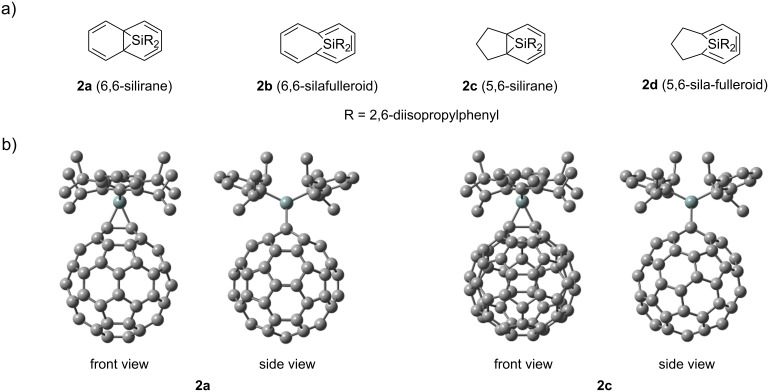
(a) Partial structures of isomers of Dip_2_SiC_60_. (b) Optimized structures of **2a** and **2c**. Hydrogen atoms are omitted for clarity.

**Figure 7:**
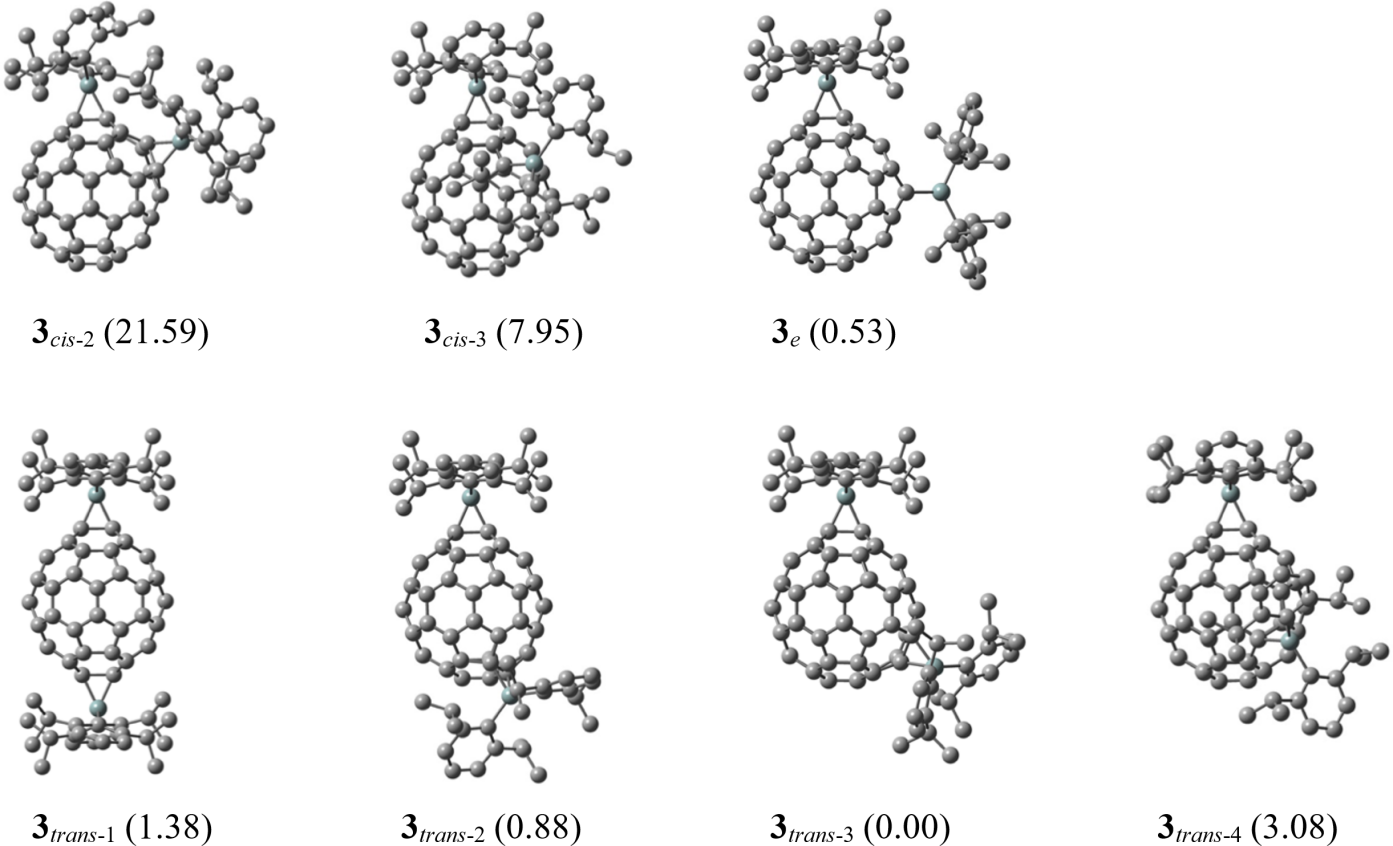
Optimized structures of **3***_cis_*_-2_, **3***_cis_*_-3_, **3***_e_*, **3***_trans_*_-1_, **3***_trans_*_-2_, **3***_trans_*_-3_, and **3***_trans_*_-4_. Values in parentheses are the relative energies in kcal/mol compared to that of **3***_trans_*_-3_. Hydrogen atoms are omitted for clarity.

Hirsch and co-workers reported the bis-functionalization of C_60_ using the Bingel–Hirsch, the Bamford–Stevens reactions, and nitrene addition, indicating that preferential formation of the *e* (*e*′ or *e*′′) isomers followed by *trans*-3 isomer when at least one of the two addends was sterically demanding [5]. The formation of **3** in the present bis-silylene addition is consistent with those earlier results. To obtain some insight into regioselectivity, we performed density functional theory (DFT) calculations using the B3LYP/6-31G(d) method [45–48]. We first compared the relative stabilities of mono-adducts (Dip_2_SiC_60_) with 6,6-silirane (**2a**), 6,6-sila-fulleroid (**2b**), 5,6-silirane (**2c**), and 5,6-sila-fulleroid (**2d**) structures (Figure 6). The optimized structure of **2a** was found to be more stable than that of **2c** by 19.23 kcal/mol. In contrast, optimization using initial structures of **2b** and **2d** afforded the structures of **2a** and **2c**, respectively. Based on these results, the optimized structures of bis-adduct isomers **3***_cis_*_-2_, **3***_cis_*_-3_, **3***_e_*, **3***_trans_*_-1_, **3***_trans_*_-2_, **3***_trans_*_-3_, and **3***_trans_*_-4_ were calculated by assuming the 6,6-silirane structures for addition sites. Calculation of **3***_cis_*_-1_ was not conducted because of its sterically clouded structure. Structural parameters around the addition sites of **3***_e_* showed rough agreement with the corresponding values of **3** obtained using the X-ray structural analysis (Table 1 and Table S1 in Supporting Information File 1). As shown in Figure 7, few differences in relative energies are apparent among **3***_e_*, **3***_trans_*_-1_, **3***_trans_*_-2_, **3***_trans_*_-3_, and **3***_trans_*_-4_, although **3***_cis_*_-2_ and **3***_cis_*_-3_ are unstable. Therefore, we next investigated the structural and electronic properties of the calculated mono-adduct **2a** to evaluate its reactivity toward **1**.

The bond lengths of the cage C–C double bonds and the π-orbital axis vector (POAV) [49] of the cage carbon atoms of **2a** are presented in Tables S2 and S3 in Supporting Information File 1. Mean values are given respectively for the identical type of bonds and atoms. The differences in the double bond lengths are negligible, but the *cis*-1, *cis*-2, and *e* bonds are slightly shorter than the other bonds. The POAV values show no marked difference except for the carbons adjacent to the addition site. Therefore, neither the double bond lengths nor the POAV values of **2a** are regarded as affecting the regioselectivity.

The frontier orbitals of **2a** were then examined to elucidate the reactivity in the second silylene addition. The ground states of silylenes are known to be singlet except for a few examples [50–51]. Singlet silylenes have n orbitals as the highest occupied molecular orbitals (HOMOs) that accommodate unshared electron pairs on the silicon atoms, while empty 3p orbitals correspond to the lowest unoccupied molecular orbitals (LUMOs). In addition, silylenes are characterized by both nucleophilic properties based on the high HOMO levels, and electrophilic properties because of the low LUMO levels [50–51]. The Mulliken charge densities of **2s** are also shown in Figure S2 (Supporting Information File 1), which shows that the charge densities are nearly zero on the cage carbon atoms except those adjacent to the addition site. The charge densities of **2a** are regarded as affecting the regioselectivity. Thus, it is suggested that the regioselectivity in the silylene addition could not be estimated by the charge densities of **2a**.

**Figure 8:**
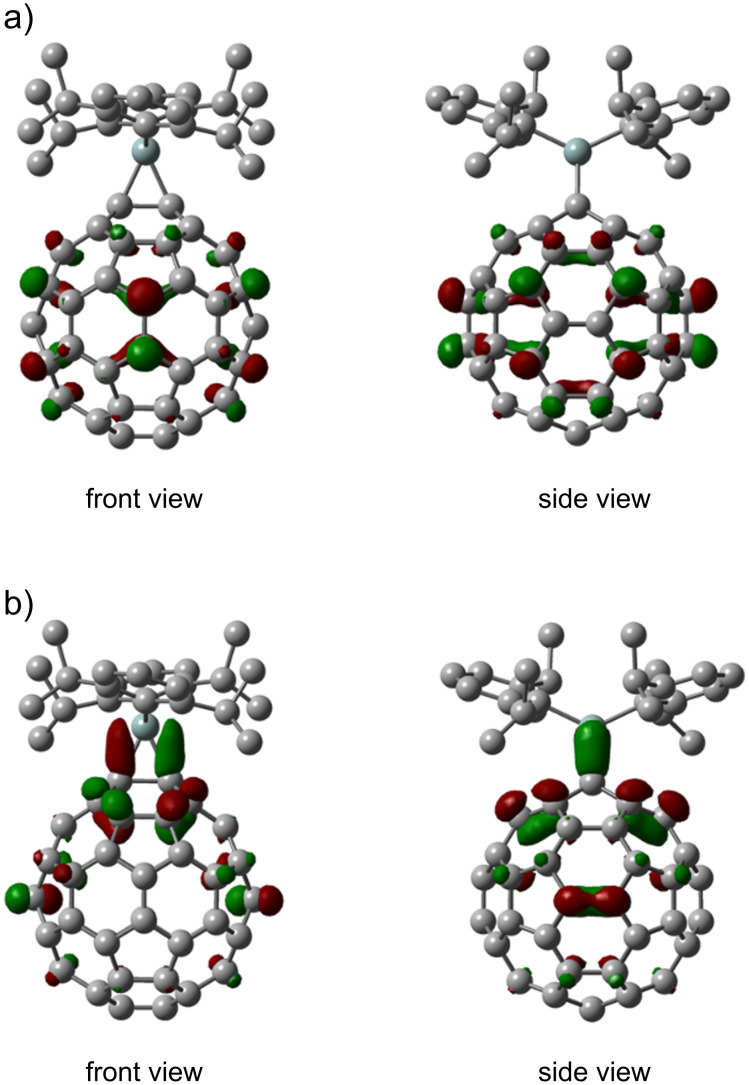
(a) LUMO and (b) HOMO of **2a** calculated at the B3LYP/6-31G(d) level. Hydrogen atoms are omitted for clarity.

The regioselectivity in the addition reaction of **1** with C_70_ was explained earlier in terms of the interaction between the HOMO of **1** and the LUMO of C_70_ [16]. The reaction mechanism of ethylene with a silylene substituted with thiolate ligands has been studied using theoretical calculations, in which the transition state was ascribed to the donor–acceptor interactions between the HOMO of the silylene and the LUMO of ethylene, and vice versa [42]. For **2a**, the LUMO is largely distributed at the *e'* bonds, followed by the *cis*-2 and *trans*-3 bonds (Figure 8). These results suggest that the formation of **3** may involve the interaction of **1** with the LUMO of **2a**. The HOMO of **2a** is observed mainly around the *cis*-1 and *e′′* bonds among the 6,6-bonds, although the *cis*-1 bond would not be susceptible to the second silylene addition because of its steric hindrance. Alternatively, the interaction of the LUMO of **1** with the HOMO of **2a** should be considered.

### Electrochemical measurements of **3**

**Figure 9:**
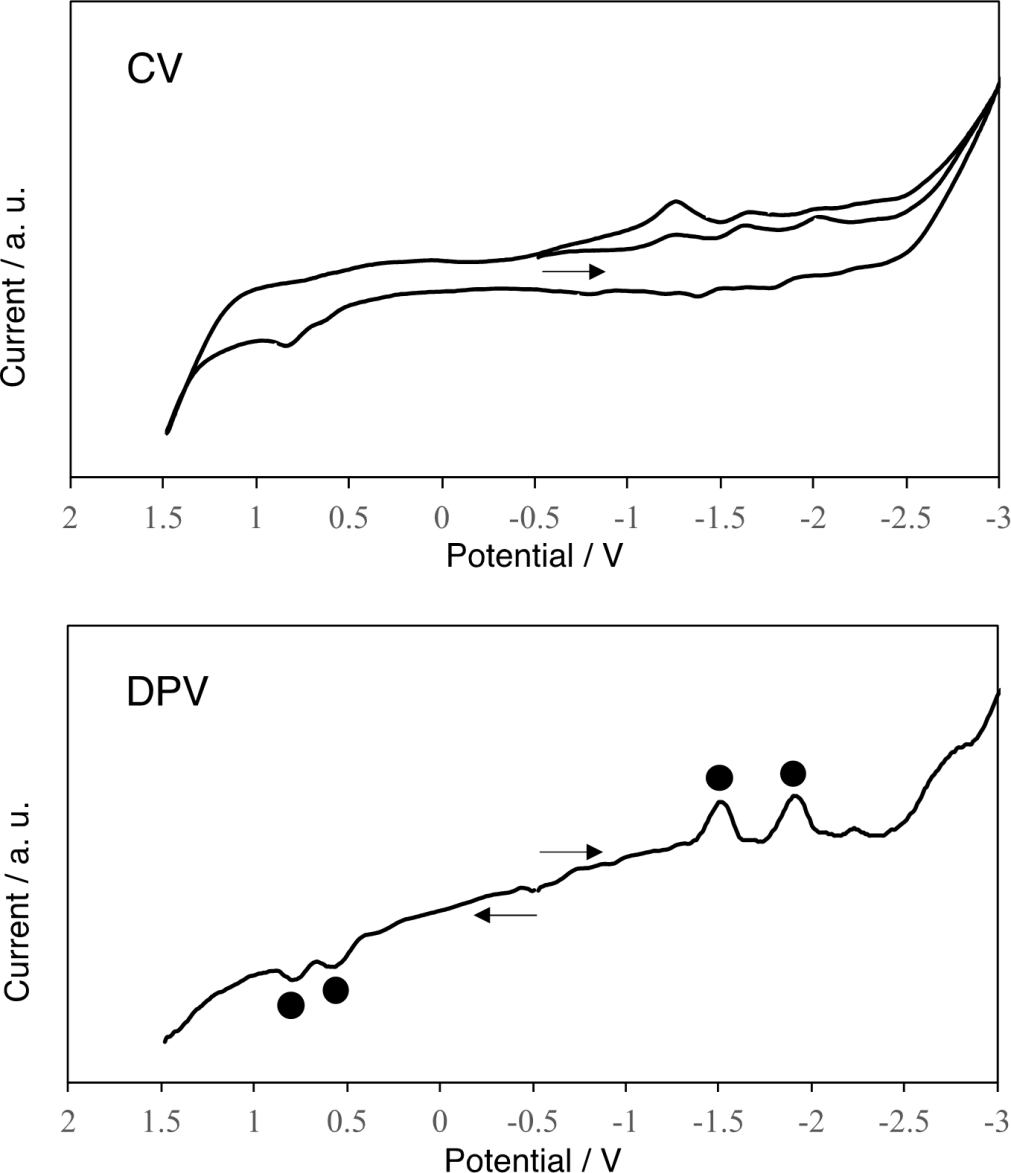
Cyclic voltammograms (CV) and differential pulse voltammograms (DPV) of **3** in *o*-dichlorobenzene containing 0.1 M (*n*-Bu)_4_NPF_6_. In DPVs, peaks of the adducts are denoted by circles. Potentials are shown in volts relative to the ferrocene/ferrocenium couple. Conditions: glassy carbon working electrode; Pt wire counter electrode; SCE reference electrode; 50 mV/s scan rate.

Cyclic voltammetry (CV) and differential pulse voltammetry (DPV) measurements were conducted to evaluate the electronic effects of the silylene addends in **3** (Figure 9). The oxidation processes of **3** were shown to be irreversible, probably because of the removal of silylene addends, whereas the electrochemically reversible behavior was observed for the reduction waves. Table 2 presents the redox potentials of **3** obtained by DPV with those of C_60_ and **2**, as reference compounds. Both the reduction (*E*^red^) and the oxidation (*E*^ox^) potentials of **3** were found to be shifted cathodically compared respectively to those of C_60_ and **2**, indicating the electron-donating effects of the two Dip_2_Si groups. It is noteworthy that the first reduction potential of **3** (*E*^red^_1_ = −1.52 V) is the most negative among those of the silylated empty fullerenes [52–56].

**Table 2:** Redox potentials^a^ (V) of C_60_, **2**, and **3**.

compound	*E* ^ox^_2_	*E* ^ox^_1_	*E* ^red^_1_	*E* ^red^_2_

C_60_		+1.32^b^	−1.15	−1.58
**2** ^b^		+0.71	−1.30	−1.71
**3**	+0.80	+0.58	−1.52	−1.92

^a^Values obtained by DPV are in volts relative to the ferrocene/ferrocenium couple. ^b^Data from ref [54].

The energies of HOMOs and LUMOs of the calculated regioisomers of **3** were compared with those of C_60_ and **2a** (Table 3). The HOMO and LUMO levels of **3**_e_ were both found to be higher than those of C_60_ and **2a**, respectively, which is qualitatively consistent with the shifts in the redox potentials of C_60_, **2**, and **3**. By contrast, **3***_cis_*_-2_, **3***_cis_*_-3_, **3***_e_*, **3***_trans_*_-1_, **3***_trans_*_-2_, **3***_trans_*_-3_, and **3***_trans_*_-4_ were found to have no significant differences in their respective HOMO and LUMO levels, as shown in Table 3.

**Table 3:** Calculated HOMO/LUMO levels (eV)^a^.

compound	HOMO	LUMO

C_60_	−5.99	−3.23
**2a**	−5.30	−2.91
**3** *_cis_*_-2_	−5.14	−2.68
**3** *_cis_*_-3_	−4.77	−2.62
**3** *_e_*	−5.01	−2.61
**3** *_trans_*_-1_	−4.99	−2.67
**3** *_trans_*_-2_	−4.97	−2.67
**3** *_trans_*_-3_	−4.90	−2.58
**3** *_trans_*_-4_	−4.86	−2.58

^a^B3LYP/6-31G(d).

## Conclusion

The bis-silylene adduct **3** was isolated by the two-fold addition of **1** to C_60_. The spectroscopic and crystallographic analyses established **3** as an *e* isomer of bis-adducts with silirane structures at the 6,6-bonds. In the DFT calculations of mono-adduct **2a**, the LUMO is distributed remarkably around the *e’* bonds, which are likely to interact with the HOMO of **1** leading to the formation of **3**. Also, the HOMO of **2a** is observed around the *cis*-1 and *e′′* bonds, although the *cis*-1 bond would be sterically protected from the second silylene addition. Alternatively, the interaction of the LUMO of **1** with *e′′* bonds of **2** should be considered. Electrochemical measurements (CV and DPV) were taken to evaluate the electronic properties of **3**. As expected, the redox potentials of **3** are shown to be shifted cathodically compared to those of C_60_ and **2** because of the electron-donating effect of the two silylene groups. These results are qualitatively consistent with the shifts in the HOMO and LUMO levels calculated respectively for C_60_, **2**, and **3**.

## Experimental

**Materials and general method:** All chemicals were reagent grade, purchased from commercial suppliers. *o*-Dichlorobenzene (ODCB) was distilled from P_2_O_5_ under vacuum before use. Toluene was distilled from benzophenone sodium ketyl under dry N_2_ prior to use. Reagents were used as purchased unless otherwise specified. The ^1^H and ^13^C NMR measurements were conducted on a JEOL ECA-500 spectrometer (JEOL Ltd.). Absorption spectra were measured using a UV-3150 spectrophotometer (Shimadzu Corp.). Cyclic voltammograms and differential pulse voltammograms were recorded on a BAS CV50W electrochemical analyzer (BAS Inc.). The reference electrode was a saturated calomel reference electrode (SCE). The glassy carbon electrode was used as the working electrode, and a platinum wire was used as the counter electrode. All potentials are referenced to the ferrocene/ferrocenium couple (Fc/Fc^+^) as the standard. (*n*-Bu)_4_NPF_6_ (0.1 M) in ODCB was used as the supporting electrolyte solution. The cyclic voltammograms were recorded using a scan rate of 50 mV/s. The differential pulse voltammograms were obtained using a pulse amplitude of 50 mV, a pulse width of 50 ms, a pulse period of 200 ms, and a scan rate of 50 mV/s.

**Synthesis of 3.** A degassed solution of Dip_2_Si(SiMe_3_) (30 mg) and C_60_ (4.6 mg) in toluene (20 mL) in a quartz tube was irradiated by a 125-W low-pressure mercury lamp for 2 h [15]. After the photolysis, **2** (22% yield) and **3** (36% yield) were isolated by flash column chromatography (SiO_2_) using mixed solvents of hexane/CH_2_Cl_2_ by changing the ratios of volumes from 10:1 to 3:1. TLC analysis (SiO_2_, hexane/CH_2_Cl_2_ 5:1) afforded *R*_f_ = 0.55 for **2** and *R*_f_ = 0.33 for **3**, respectively. Data for compound **3**: ^1^H NMR (CDCl_3_/CS_2_ 3:1) δ 7.61 (t, *J* = 7.5 Hz, 1H), 7.56 (t, *J* = 7.5 Hz, 1H), 7.51 (t, *J* = 7.5 Hz, 2H), 7.41 (d, *J* = 7.5 Hz, 2H), 7.35 (d, *J* = 7.5 Hz, 2H), 7.31 (d, *J* = 7.5 Hz, 2H), 7.26 (d, *J* = 7.5 Hz, 2H), 4.04 (sept, *J* = 7.0 Hz, 2H), 4.03 (sept, *J* = 7.0 Hz, 2H), 3.95 (sept, *J* = 7.0 Hz, 2H), 3.52 (sept, *J* = 7.0 Hz, 2H), 1.43 (d, *J* = 7.0 Hz, 6H), 1.40 (d, *J* = 7.0 Hz, 6H), 1.371 (d, *J* = 7.0 Hz, 6H), 1.365 (d, *J* = 7.0 Hz, 6H), 1.31 (d, *J* = 7.0 Hz, 6H), 1.29 (d, *J* = 7.0 Hz, 6H), 1.17 (d, *J* = 7.0 Hz, 6H), 1.06 (d, *J* = 7.0 Hz, 6H); ^13^C NMR (CDCl_3_/CS_2_ 3:1) δ 160.16 (s, 2C), 158.38 (s, 2C), 158.33 (s, 2C), 158.29 (s, 2C), 158.18 (s, 2C), 157.12 (s, 2C), 155.78 (s, 2C), 154.87 (s, 2C), 148.96 (s, 1C), 148.73 (s, 2C), 148.56 (s, 2C), 147.66 (s, 2C), 147.48 (s, 2C), 147.22 (s, 2C), 146.69 (s, 1C), 146.26 (s, 2C), 145.51 (s, 2C), 145.37 (s, 2C), 145.03 (s, 2C), 144.84 (s, 2C), 144.71 (s, 2C), 144.65 (s, 4C), 144.51 (s, 2C), 144.23 (s, 2C), 142.90 (s, 2C), 142.43 (s, 2C), 141.62 (s, 2C), 139.22 (s, 2C), 139.05 (s, 2C), 138.21 (s, 2C), 138.15 (s, 2C), 133.37 (s, 2C), 132.76 (d, 1C), 132.56 (d, 2C), 131.71 (d, 1C), 125.43 (s, 1C), 124.79 (d, 2C), 124.71 (d, 2C), 124.66 (s, 1C), 124.49 (d, 2C), 124.35 (d, 2C), 123.85 (s, 2C), 71.92 (s, 2C), 70.39 (s, 1C), 69.12 (s, 1C), 37.59 (d, 2C), 37.35 (d, 2C), 37.24 (d, 2C), 37.11 (d, 2C), 28.55 (q, 2C), 27.86 (q, 2C), 27.57 (q, 2C), 27.44 (q, 2C), 25.61 (q, 2C), 25.25 (q, 2C), 25.10 (q, 2C), 24.11 (q, 2C); UV–vis (CH_2_Cl_2_) λ_max_ 515 nm.

**X-ray crystallography of 3:** Black plate crystals suitable for X-ray diffraction were obtained using the liquid–liquid bilayer diffusion method with solutions of **3** in CS_2_ using hexane as a poor solvent at 0 °C. Single-crystal X-ray diffraction data of **3** were collected on a Rigaku Oxford Diffraction XtaLAB Synergy R DW system with a HyPix detector equipped with a nitrogen-gas flow low-temperature apparatus providing a constant temperature at 100 K. Crystal data for C_60_(Dip_2_Si)_2_: C_108_H_68_Si_2_: *M*_r_ = 1421.87, black plate, 0.11 × 0.16 × 0.025 mm, λ = 0.71073 Å, monoclinic, space group *P*2_1_/*n* (no. 14), *a* = 18.3631(3), *b* = 13.6696 (2), *c* = 27.6360 (5) Å, β = 92.857 (2)°, *V* = 6928.5(2) Å^3^, *T* = 99.9(2) K, *Z* = 4, 67750 reflections measured, 19176 unique (*R*_int_ = 0.0321), which were used for all calculations, 2θ_max_ = 63.262; min/max transmission = 0.921/1.000 (absorption correction was applied by multi-scan method); The structure was solved using a direct method using olex2.solve 1.5-ac5-018 [57] and was refined with SHELXL-2018 [58]. The final *wR*(F_2_) was 0.1283 (all data), conventional *R*_1_ = 0.0493 computed for 14459 reflections with *I* > 2σ(*I*) using 1007 parameters with 0 restraints. Crystallographic computations were performed with Olex2 [59]. CCDC 2311474 (**3**) contains the supplementary crystallographic data for this paper, and is obtainable free of charge from the Cambridge Crystallographic Data Centre.

**Computational method.** All calculations were conducted using the Gaussian 16 program [60]. Optimized structures were obtained at the B3LYP [45–47] level of theory using basis sets of 6-31G(d) [48].

## Supporting Information

File 1 Structural data of **2a** and **3**_e_ obtained by DFT calculations, and Cartesian coordinates of optimized structures.

File 2 Crystallographic information file of **3**.

## Data Availability

All data that supports the findings of this study is available in the published article and/or the supporting information to this article.
