# Supplementary material for: Two-fold addition reaction of silylene to C60: structural and electronic properties of a bis-adduct
Source: Beilstein J Org Chem. 2024 May 22;20:1179–88. doi: 10.3762/bjoc.20.100 (PMC11181185; doi:10.3762/bjoc.20.100)
Supplement: File 1 — Structural data of 2a and 3e obtained by DFT calculations, and Cartesian coordinates of optimized structures. [file Beilstein_J_Org_Chem-20-1179-s001.pdf]

BEILSTEIN JOURNAL OF ORGANIC CHEMISTRY

Supporting Information

for

Two-fold addition reaction of silylene to C_{60}: structural and electronic properties of a bis-adduct

Masahiro Kako, Masato Kai, Masanori Yasui, Michio Yamada, Yutaka Maeda and Takeshi Akasaka

Beilstein J. Org. Chem. **2024,** *20,* 1179–1188. doi:10.3762/bjoc.20.100

Structural data of 2a and 3_e obtained by DFT calculations, and Cartesian coordinates of optimized structures

License and Terms: This is a supporting information file under the terms of the Creative Commons Attribution License (https://creativecommons.org/licenses/by/4.0). Please note that the reuse, redistribution and reproduction in particular requires that the author(s) and source are credited and that individual graphics may be subject to special legal provisions.
The license is subject to the *Beilstein Journal of Organic Chemistry* terms and conditions: (https://www.beilstein-journals.org/bjoc/terms)

Table of contents

Figure S1. Optimized structure of $\mathbf{3}_e$. S2

Table S1. Selected bond lengths and angles of $\mathbf{3}_e$. S2

Table S2. Bond lengths of **2a**. S3

Table S3. POAV values of **2a**. S3

Figure S2. The Mulliken charge distribution of **2a**. S4

Table S4. Cartesian coordinates of optimized structures. S5

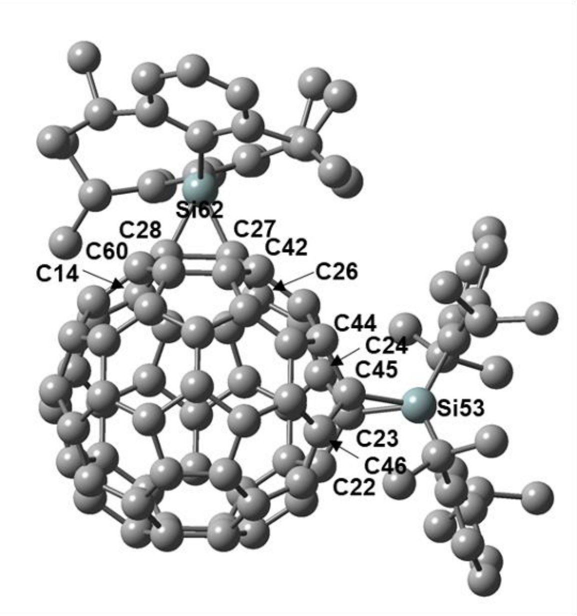

Figure S1: Optimized structure of **3**e. Hydrogen atoms are omitted for clarity.

Table S1. Selected bond lengths and angles of **3**e.

bond length/Å		bond angle/°	
C45–C23	1.618	C23–C45–Si53	64.94
Si53–C45	1.906	C45–Si53–C23	50.19
C23–Si53	1.908	Si53–C23–C45	64.86
C45–C46	1.500		
C45–C44	1.503		
C23–C24	1.503		
C23–C22	1.502		
C27–C28	1.623	C28–C27–Si62	64.56
Si62–C27	1.913	C27–Si62–C28	50.33
C28–Si62	1.905	Si62–C28–C27	65.10
C42–C27	1.499		
C27–C26	1.502		
C60–C28	1.501		
C28–C14	1.499		

Table S2. Bond lengths[a] of **2a**[b].

bond	bond length/Å
cis-1	1.384
cis-2	1.394
cis-3	1.401
e'	1.395
e''	1.394
trans-4	1.396
trans-3	1.396
trans-2	1.397
trans-1	1.396

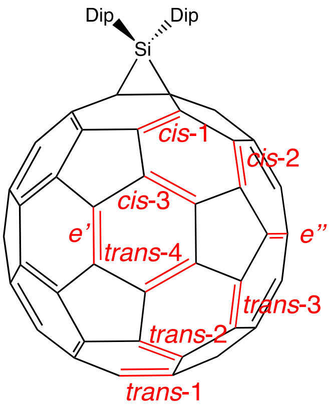

[a]Mean values are given for the identical type of double bonds.

[b]B3LYP/6-31G(d).

Table S3. POAV[a] values of **2a**[b].

carbon atom	POAV/°	carbon atom	POAV/°
a	9.15	j	11.72
b	11.58	k	11.67
c	10.81	l	11.74
d	11.18	m	11.60
e	11.83	n	11.65
f	11.27	o	11.66
g	12.00	p	11.60
h	11.65	q	11.60
i	11.69		

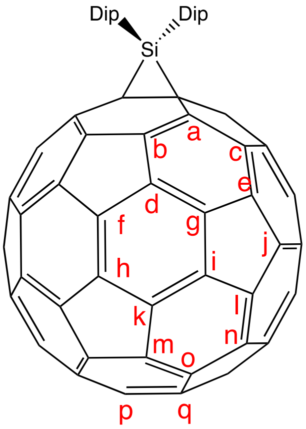

[a] Mean values are given for the identical type of atoms.

[b] B3LYP/6-31G(d).

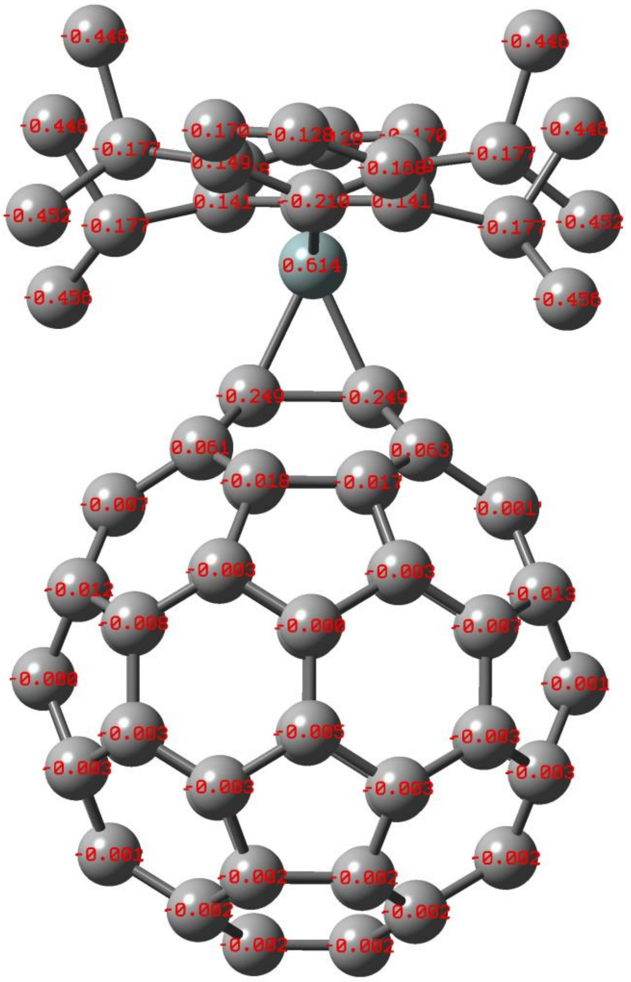

Figure S2. The Mulliken charge distribution of **2a**. The hemisphere of the C_{60} carbon cage and hydrogen atoms are not shown for clarity.

Table S4. Cartesian coordinates of optimized structures.

2a

C	-2.31523200	0.85758600	3.45395400
C	-1.14085700	0.00476000	3.35612800
C	-0.01568300	0.44597200	2.64995700
C	-0.01917700	1.75381200	2.02754400
C	-1.14185600	2.58210300	2.13636300
C	-2.31561000	2.11837700	2.85865600
C	-3.48875900	0.00716500	3.35698200
C	-3.03331000	-1.36526200	3.19996900
C	-1.58158500	-1.35960300	3.20353300
C	-0.87383400	-2.21318400	2.34735200
C	0.74420300	-0.44937100	1.81078800
C	0.73337200	1.66969000	0.80108300
C	0.30009700	2.37558300	-0.30818900
C	-0.87578400	3.21163000	-0.21790600
C	-1.58177100	3.33149300	0.98622300
C	-3.03315300	3.33570100	0.98067200
C	-3.48882700	2.58524400	2.14046900
C	-4.61678200	1.76823800	2.04432900
C	-4.61667200	0.45285500	2.66504700
C	-3.72795500	-2.23528200	2.35701500
C	-4.90171700	-1.77321100	1.64161600
C	-5.33811500	-0.45500600	1.79083400
C	-5.78601200	0.29768300	0.63121000
C	-5.33822300	1.67127700	0.78764300
C	-4.90160800	2.39405500	-0.32490700
C	-3.72745000	3.23918600	-0.22692800
C	-2.99550900	3.13416700	-1.47959800
C	-1.60068700	3.11584000	-1.47215500
C	1.43136900	0.34332200	0.73501300
C	-1.14089700	-0.00473400	-3.35619900
C	-0.01569700	-0.44590200	-2.65004200
C	-0.01913200	-1.75374300	-2.02763200
C	-1.14178000	-2.58207700	-2.13643600
C	-2.31556200	-2.11839600	-2.85871500
C	-3.48879900	-0.00723000	-3.35702700
C	-3.03340100	1.36521400	-3.20002000
C	-1.58167600	1.35961200	-3.20360100
C	-0.87394800	2.21321800	-2.34742700
C	0.30557100	1.75226300	-1.64896700
C	0.74417100	0.44947100	-1.81088300
C	0.73342400	-1.66958900	-0.80117900
C	0.30019500	-2.37550500	0.30809700
C	-0.87565600	-3.21159700	0.21782900
C	-1.58165200	-3.33148700	-0.98629100
C	-3.03303500	-3.33575000	-0.98072300
C	-3.48875100	-2.58530900	-2.14051400
C	-4.61673700	-1.76834800	-2.04436000
C	-4.61668500	-0.45296500	-2.66507800
C	-3.72807000	2.23520700	-2.35705800
C	-4.90180500	1.77309000	-1.64164400
C	-5.33815300	0.45486800	-1.79085700
C	-5.78600700	-0.29783900	-0.63122800
C	-5.33816700	-1.67141500	-0.78766600
C	-4.90151000	-2.39417600	0.32487900
C	-3.72732100	-3.23926100	0.22688600
C	-2.99536900	-3.13421400	1.47954700
C	-1.60054800	-3.11583400	1.47208700
C	1.43138000	-0.34319900	-0.73512200
C	0.30565700	-1.75218200	1.64887700
C	-2.31523900	-0.85760500	-3.45401200
Si	3.15918500	0.00004400	-0.00000100
C	3.99375500	1.57497500	-0.72536500
C	4.49415200	1.51009400	-2.05867300
C	4.00064100	2.84661200	-0.06773700
C	4.89882300	2.67546400	-2.72020900
C	4.42620200	3.98067200	-0.77310100
C	4.85297700	3.91042900	-2.09161700
H	5.25878900	2.60968700	-3.74271800

H	4.42693300	4.94303100	-0.27152400
H	5.16325400	4.80959700	-2.61760900
C	3.99375400	-1.57485200	0.72538500
C	4.49378400	-1.51001500	2.05882900
C	4.00096000	-2.84643600	0.06765300
C	4.89862500	-2.67535600	2.72031100
C	4.42670100	-3.98046000	0.77297000
C	4.85326900	-3.91025300	2.09155400
H	5.25840900	-2.60959100	3.74288700
H	4.42768000	-4.94278000	0.27131500
H	5.16371700	-4.80939500	2.61749100
C	3.66835100	3.08468700	1.40743100
H	3.17329200	2.19532000	1.80493700
C	4.97437500	3.30773600	2.20173300
H	5.45081200	4.24713400	1.89852000
H	4.76699600	3.37069500	3.27697800
H	5.69822300	2.50607700	2.03508200
C	2.72214500	4.27860000	1.65465200
H	1.83993800	4.24383700	1.01245300
H	2.38563200	4.27364700	2.69759200
H	3.22722400	5.23609200	1.48487800
C	4.65828100	0.20534000	-2.82962500
H	4.35117600	-0.61524600	-2.17402800
C	6.13852400	-0.03846900	-3.18906200
H	6.25528900	-0.99221800	-3.71427700
H	6.52343000	0.74853500	-3.84656700
H	6.76837400	-0.05966700	-2.29280200
C	3.77175900	0.14149600	-4.08755900
H	3.87087600	-0.83552400	-4.57530800
H	2.71671100	0.29719100	-3.84374300
H	4.06262100	0.90575200	-4.81745900
C	3.66852300	-3.08460300	-1.40746800
H	3.17344300	-2.19526000	-1.80500400
C	4.97440900	-3.30785300	-2.20194100
H	4.76687300	-3.37069500	-3.27716100
H	5.69845200	-2.50635200	-2.03534400
H	5.45070600	-4.24736800	-1.89887400
C	2.72219300	-4.27848600	-1.65438000
H	1.83998400	-4.24343200	-1.01220100
H	2.38569600	-4.27379300	-2.69732700
H	3.22716300	-5.23598200	-1.48430500
C	4.65753100	-0.20535100	2.83000600
H	4.35008200	0.61532400	2.17466600
C	6.13772600	0.03866700	3.18946400
H	6.76764300	0.05970300	2.29324700
H	6.25440600	0.99251800	3.71450300
H	6.52260300	-0.74820600	3.84715500
C	3.77103000	-0.14202500	4.08798900
H	3.86974600	0.83499100	4.57582500
H	2.71603800	-0.29811800	3.84415400
H	4.06222100	-0.90621800	4.81782000

2c

C	1.86230714	0.93924806	-3.40165922
C	0.50114605	0.97606707	-2.90089818
C	0.13195503	1.94278214	-1.98093312
C	1.08813711	2.93646520	-1.54709809
C	2.39763720	2.91799419	-2.03607113
C	2.79488022	1.89486411	-2.98430119
C	2.25725717	-0.45230905	-3.51919323
C	1.14188408	-1.27520210	-3.08167420
C	0.04896301	-0.40895403	-2.70107217
C	-0.78101506	-0.72904805	-1.63658910
C	-0.74970504	1.61824613	-0.86280504
C	0.82818909	3.17444322	-0.14601699
C	1.88854017	3.40652623	0.71632007
C	3.25075927	3.39115222	0.22218004
C	3.50513728	3.15037620	-1.13231906
C	4.59483735	2.26944813	-1.51796609
C	4.15416332	1.48931807	-2.66564817

C	4.53301433	0.15316098	-2.78749818
C	3.56516126	-0.83902009	-3.22552821
C	1.37755008	-2.46053319	-2.38530015
C	2.73828218	-2.86323623	-2.07913213
C	3.81082326	-2.06749118	-2.48614715
C	4.92529335	-1.83226617	-1.58658209
C	5.36967537	-0.46365308	-1.76815510
C	5.79183245	0.28508397	-0.66755403
C	5.39365742	1.67853108	-0.53983702
C	5.14127639	1.94395910	0.86727309
C	4.08887532	2.78110717	1.24061211
C	-0.31745000	2.33970017	0.25628404
C	2.39192018	0.43516901	3.52801827
C	1.08148709	0.81358205	3.21731525
C	0.12207601	-0.17107301	2.76726022
C	0.49823403	-1.49915111	2.65395621
C	1.85784512	-1.90033215	2.96359923
C	4.15009929	-0.98883610	2.88561123
C	4.59120034	0.38589999	2.70093622
C	3.50092927	1.26145507	3.09878825
C	3.24803626	2.43036615	2.37358519
C	1.88619916	2.80911919	2.05503317
C	0.82236008	2.01601514	2.46023720
C	-0.76627805	0.44664604	1.78210815
C	-1.45615610	-0.37387001	0.77260408
C	-0.76809906	-1.68739711	0.53288806
C	0.05507399	-2.26738416	1.48413013
C	1.14184806	-3.13565124	1.09741710
C	2.25408014	-2.91700623	2.00604416
C	3.56206124	-2.95909724	1.52386013
C	4.52971831	-1.97144318	1.97199516
C	5.39189441	0.71893301	1.60908614
C	5.79095941	-0.30627807	0.65699107
C	5.36799836	-1.62533716	0.83337108
C	4.92433634	-2.40315621	-0.30785900
C	3.80868126	-3.22897926	0.11558603
C	2.73735617	-3.45559927	-0.75103103
C	1.37640507	-3.41164025	-0.24932400
C	0.54237202	-2.78261720	-1.24988207
C	-0.50505605	-1.92122413	-0.86792004
C	-0.32664200	1.75990513	1.57284514
C	-1.45678110	0.33316604	-0.80840604
C	2.78996220	-0.95372609	3.39907327
Si	-3.15179122	0.00282003	-0.00963598
C	-3.95485227	1.56191814	0.77789308
C	-4.44322430	1.43555414	2.11151117
C	-3.94519425	2.86587824	0.18861004
C	-4.81533632	2.57184322	2.83939423
C	-4.33542727	3.96959332	0.95945609
C	-4.74678630	3.83784632	2.27817718
H	-5.16902935	2.45849822	3.85997730
H	-4.32155327	4.95712440	0.50966206
H	-5.03002532	4.71395938	2.85584423
C	-4.07043430	-1.52024608	-0.75226603
C	-4.64342234	-1.37617106	-2.05153312
C	-4.13084832	-2.80972017	-0.13467299
C	-5.19697339	-2.48155613	-2.70775917
C	-4.71651036	-3.87782024	-0.82974104
C	-5.23372240	-3.73081122	-2.10773413
H	-5.60761140	-2.35784612	-3.70519825
H	-4.76280438	-4.85221831	-0.35400700
H	-5.66758041	-4.58113428	-2.62750817
C	-3.63890523	3.17102926	-1.27925707
H	-3.16216620	2.29643320	-1.72857211
C	-4.95987832	3.44644929	-2.03143312
H	-5.41403233	4.37855236	-1.67595610
H	-4.77758831	3.55343230	-3.10779020
H	-5.69160538	2.64904124	-1.88138911
C	-2.68656815	4.36623434	-1.49160908
H	-1.79475809	4.29722533	-0.86616904

H	-2.36707313	4.40259334	-2.53939216
H	-3.18057718	5.31993239	-1.27412307
C	-2.67300022	-4.36096229	1.28636112
H	-1.87708016	-4.25716029	0.54584406
H	-2.20802819	-4.45741830	2.27381419
H	-3.20111827	-5.29911837	1.08217810
C	-4.69958033	-0.05258396	-2.80715518
H	-4.34709630	0.73662909	-2.13630913
C	-6.14381041	0.31166907	-3.20644621
H	-6.80098447	0.35912008	-2.33104514
H	-6.16852442	1.28447314	-3.70783724
H	-6.56686846	-0.42289997	-3.89978326
C	-3.77546727	-0.05097497	-4.04033927
H	-3.78639526	0.93195810	-4.52631930
H	-2.74245519	-0.28838300	-3.76923225
H	-4.10510330	-0.79068602	-4.77932332
C	-4.63964233	0.09374505	2.80918522
H	-4.34077831	-0.69816101	2.11485317
C	-3.64019928	-3.15709720	1.27349111
H	-3.09976024	-2.30053014	1.68268714
C	-6.13047842	-0.13537396	3.13430925
H	-6.27579843	-1.10584203	3.62068928
H	-6.51105646	0.63311510	3.81580630
H	-6.74447146	-0.10889895	2.22733118
C	-3.77195726	-0.05684897	4.07261331
H	-4.05529028	0.67309408	4.83956637
H	-3.89951728	-1.05608304	4.50553635
H	-2.71072219	0.08735703	3.84945930
C	-4.83241737	-3.46093721	2.20624018
H	-4.48680035	-3.59864722	3.23795026
H	-5.57631544	-2.66091214	2.19501418
H	-5.33965239	-4.38332927	1.90122216

3cis-2

C	0.69882500	-3.19204400	-0.54831900
C	1.70315100	-3.78930000	0.29401000
C	2.99633500	-4.03375900	-0.18145300
C	3.34064700	-3.63936800	-1.52631100
C	2.37168400	-3.01417200	-2.32279900
C	1.03369100	-2.79221900	-1.82313100
C	-0.31571600	-2.48199600	0.29925200
C	0.38229500	-2.39707500	1.62173900
C	1.50461300	-3.29486200	1.63968800
C	2.60810300	-3.07070500	2.47422800
C	4.14159400	-3.78439300	0.67833300
C	4.70170400	-3.13756500	-1.50946000
C	5.05266900	-2.04356900	-2.30464300
C	4.05236000	-1.40551400	-3.14098900
C	2.73916900	-1.87534600	-3.14175800
C	1.62683000	-0.94218700	-3.13393100
C	0.57768900	-1.47810800	-2.31848000
C	-0.21519200	-0.63202400	-1.54604000
C	-0.86968600	-1.06497000	-0.26289100
C	0.43455400	-1.24177300	2.37919200
C	-0.05046200	0.04490300	1.85271200
C	-0.61344700	0.14456600	0.61709900
C	-0.44753600	1.38762800	-0.23340700
C	0.01647200	0.78392700	-1.54371300
C	1.06070100	1.30002300	-2.31307200
C	1.86747500	0.43658500	-3.12998400
C	5.20056100	-3.23052000	-0.14587500
C	6.09396800	-0.31775300	1.73119400
C	5.31635700	0.52630300	2.53000500
C	4.22224000	2.82834900	-0.14035300
C	5.83902300	-1.74611200	1.72302400
C	4.81652800	-2.27617700	2.51467200
C	4.00458600	-1.39763600	3.33568400
C	4.24912800	-0.02332900	3.34410000
C	3.13848500	0.91623300	3.31823500
C	3.52089500	2.04208400	2.50719900

C	2.56130200	2.64933700	1.68408100
C	2.92446900	3.04046200	0.33810200
C	1.95853300	2.34122200	-1.78216700
C	1.77963500	2.82288400	-0.50559900
C	0.57705100	2.52745300	0.33065100
C	1.18954700	2.20948600	1.66630900
C	0.83913500	1.09083000	2.40341600
C	1.82264300	0.44223500	3.23963600
C	1.57172300	-0.98583500	3.22712700
C	2.63793900	-1.89090500	3.29889900
C	6.03144700	-2.23036300	0.36540200
C	3.94971900	-3.31496800	1.97898400
Si	-1.20004400	3.16304500	0.08448500
C	5.91979400	-1.00619200	-1.77390500
C	6.40271400	-1.09885200	-0.46472200
C	6.01397200	1.31383400	-0.12000500
C	6.44546900	0.08262600	0.37841300
C	5.21078700	2.19208400	0.71274100
C	4.86442300	1.80726400	2.00868900
C	5.46486500	0.27225500	-2.29116200
C	5.51495800	1.41149500	-1.48297000
C	3.23672800	0.92171200	-3.12549500
C	4.30696000	0.02716800	-3.13246300
C	3.28719000	2.10157200	-2.28716200
C	4.41050100	2.35053100	-1.48868800
Si	-2.16765200	-2.52096600	-0.15230500
C	-1.81599300	4.27597900	-1.38529200
C	-1.04022000	5.18294700	-2.17693300
C	-3.22680400	4.26151800	-1.60285100
C	-1.67907200	6.01506600	-3.10710000
C	-3.81255500	5.11221600	-2.54963600
C	-3.05114300	5.99238900	-3.30037400
H	-1.07613300	6.69596900	-3.69957200
H	-4.88778800	5.08239000	-2.69660100
H	-3.51917000	6.64967000	-4.02864900
C	-1.98531900	3.69876100	1.76655200
C	-2.86161600	2.93971400	2.59631700
C	-1.66015300	5.02766900	2.18068500
C	-3.40281000	3.52515300	3.75084200
C	-2.21526300	5.55685800	3.35231300
C	-3.09318900	4.82079200	4.13308500
H	-4.07402900	2.94091700	4.37251300
H	-1.95146000	6.56520100	3.65669400
H	-3.52256600	5.24798500	5.03569500
C	-3.53722200	-2.78397600	1.20191100
C	-4.89105000	-2.46248000	0.86338900
C	-3.30217500	-3.33732400	2.50498900
C	-5.91345700	-2.60442200	1.80982000
C	-4.36303000	-3.43353000	3.41912700
C	-5.65574500	-3.05919900	3.09400700
H	-6.93276500	-2.35975500	1.53107700
H	-4.16510600	-3.83596900	4.40744200
H	-6.45790300	-3.14695600	3.82220900
C	-2.43430200	-3.42194000	-1.83600700
C	-2.32542200	-4.84571000	-1.80495400
C	-2.69468000	-2.79427600	-3.09637500
C	-2.43721000	-5.58867700	-2.98655600
C	-2.78890600	-3.58878100	-4.24850100
C	-2.65668100	-4.96897900	-4.20741800
H	-2.35007900	-6.67043400	-2.94630600
H	-2.97483900	-3.10887400	-5.20432700
H	-2.73252100	-5.55614200	-5.11903700
C	-4.20359800	3.35873300	-0.85492300
H	-3.64340200	2.72908600	-0.15389200
C	0.48166900	5.31455900	-2.15656100
H	0.87542300	4.68111400	-1.36437000
C	0.98734600	6.74307300	-1.87237500
H	2.08310400	6.74302200	-1.83821600
H	0.68561800	7.45076800	-2.65260400
H	0.62242300	7.12609700	-0.91691700

C	1.07403200	4.82326500	-3.49459600
H	2.16814700	4.80554200	-3.44813700
H	0.72716000	3.81722100	-3.74666600
H	0.78488200	5.49032600	-4.31521700
C	-5.20747800	4.19128400	-0.03209500
H	-4.69305200	4.80867400	0.71060600
H	-5.79099100	4.85561600	-0.67857100
H	-5.91623500	3.54485200	0.49569700
C	-4.92595200	2.40236700	-1.82101900
H	-4.20759100	1.79216800	-2.37830500
H	-5.59248000	1.73120900	-1.27165300
H	-5.53503800	2.94877200	-2.54949800
C	-0.70548900	5.95698200	1.43011700
H	-0.36203600	5.45635800	0.52032600
C	-1.41822100	7.25238100	0.99275800
H	-0.72906700	7.91916900	0.46404100
H	-2.25867900	7.03469600	0.32575100
H	-1.80628200	7.80544600	1.85476100
C	0.55744200	6.26038800	2.26120300
H	1.07367400	5.33835700	2.54793600
H	1.25598700	6.87657000	1.68284700
H	0.31316400	6.80734800	3.17889800
C	-3.24415700	1.47560400	2.38000000
H	-2.75372400	1.11069500	1.47229800
C	-2.75353000	0.61148400	3.56122900
H	-2.90439700	-0.44980900	3.34075700
H	-1.69470400	0.77722800	3.77348800
H	-3.31638300	0.84182600	4.47316500
C	-4.76128600	1.26463400	2.21539900
H	-5.15399900	1.77168300	1.33154000
H	-4.97991200	0.19616200	2.12456200
H	-5.31478400	1.63948500	3.08364400
C	-2.92894000	-1.29507100	-3.33527700
H	-2.76167200	-0.75719700	-2.39577100
C	-1.98811800	-3.93259100	3.01716800
H	-1.22657200	-3.81569400	2.25075100
C	-1.47835200	-3.23571000	4.29450500
H	-0.48547400	-3.61679700	4.55872000
H	-1.39893000	-2.15379500	4.16647600
H	-2.14112500	-3.42751800	5.14612400
C	-2.11960200	-5.44803900	3.28417700
H	-2.81168000	-5.65449400	4.10841600
H	-2.48189300	-5.98788500	2.40585700
H	-1.14279900	-5.86222200	3.56076100
C	-4.37454800	-1.01380700	-3.80457300
H	-4.56797600	-1.49793700	-4.76823500
H	-5.12774300	-1.37129900	-3.10304200
H	-4.52218000	0.06319200	-3.94390900
C	-1.98320500	-0.69873200	-4.40374400
H	-2.03672000	0.39572300	-4.37963000
H	-0.94466900	-0.99247400	-4.25584100
H	-2.28149300	-1.01503900	-5.40997700
C	-5.34434000	-2.06461900	-0.53900300
H	-4.46998600	-1.71640800	-1.09785600
C	-5.92501200	-3.29764300	-1.26566000
H	-5.18731800	-4.09835900	-1.35830300
H	-6.78671100	-3.68873400	-0.71204500
H	-6.26662600	-3.03651500	-2.27349700
C	-6.37658100	-0.92139500	-0.55979800
H	-6.53735200	-0.57943900	-1.58734800
H	-7.35132500	-1.24446000	-0.17858200
H	-6.04844400	-0.06785500	0.03791000
C	-2.12510000	-5.65756400	-0.52350600
H	-1.97915200	-4.96410200	0.31164600
C	-0.87253000	-6.55424700	-0.57639300
H	-0.71443400	-7.03797900	0.39459200
H	-0.97592300	-7.34816000	-1.32464800
H	0.02374300	-5.97697300	-0.81916300
C	-3.38000300	-6.49727500	-0.20704400
H	-3.60992500	-7.18484000	-1.02866600

H	-3.22642900	-7.10219300	0.69372400
H	-4.25726600	-5.86306600	-0.04376700

3cis-3

C	-1.81334300	4.17066800	-2.70914100
C	-1.47353600	5.34157200	-1.92287800
C	-0.14362500	5.76664000	-1.84427700
C	0.89435700	5.03923300	-2.54876900
C	0.56608200	3.91165500	-3.30810100
C	-0.81632100	3.47085600	-3.38833200
C	-2.85647800	3.44473100	-1.99786800
C	-3.16058200	4.16129800	-0.78570500
C	-2.30633900	5.33560200	-0.73074600
C	-1.77858200	5.76103300	0.48943300
C	0.40206200	6.21186900	-0.57126100
C	2.07936600	5.02582700	-1.70831600
C	2.88149700	3.88457700	-1.65062200
C	2.53487800	2.71100900	-2.42696200
C	1.40586300	2.72953700	-3.25229300
C	0.54784200	1.56967300	-3.29050100
C	-0.81727100	2.01671900	-3.38130900
C	-1.80560100	1.31975000	-2.68050500
C	-2.84852100	2.04471700	-1.98407300
C	-3.43377600	3.43855900	0.38270600
C	-3.44776000	1.99375600	0.37367700
C	-3.18617500	1.30247000	-0.79557400
C	-2.51779200	-0.04238700	-0.83841700
C	-1.50277800	0.13042600	-1.92432600
C	-0.18325700	-0.28435900	-1.81875500
C	0.85350000	0.45067400	-2.50134200
C	1.77843300	5.76106700	-0.48949100
C	1.81322700	4.17072500	2.70909500
C	0.81622000	3.47089700	3.38829100
C	1.50274700	0.13047000	1.92431000
C	2.51776200	-0.04232800	0.83840100
C	1.47339600	5.34161600	1.92282300
C	0.14347600	5.76665600	1.84422000
C	-0.89449100	5.03923300	2.54871700
C	-0.56619300	3.91166700	3.30805700
C	-1.40594900	2.72953100	3.25225700
C	-0.54790300	1.56968500	3.29047300
C	-0.85353700	0.45067300	2.50132200
C	0.18323400	-0.28434400	1.81874100
C	1.87479500	-0.55487300	-0.57340100
C	0.38525700	-0.81112500	-0.56449100
C	-0.38526800	-0.81113000	0.56448000
C	-1.87481100	-0.55490800	0.57338700
C	-2.04922700	0.41963100	1.69844800
C	-2.86811500	1.53426500	1.65205100
C	-2.53496300	2.71097300	2.42692600
C	-2.88160600	3.88452800	1.65057800
C	-2.07950000	5.02579600	1.70826400
C	2.30619800	5.33565500	0.73069200
C	-0.40222000	6.21186500	0.57120100
Si	-3.28216100	-1.63884600	-0.13670700
C	3.43367500	3.43862800	-0.38274700
C	3.16046700	4.16137000	0.78565900
C	2.84844900	2.04479100	1.98404200
C	2.85637700	3.44480500	1.99782800
C	1.80554400	1.31980600	2.68048000
C	0.81720000	2.01675900	3.38127900
C	3.44769000	1.99382600	-0.37370800
C	3.18611800	1.30254300	0.79554900
C	2.04919100	0.41966100	-1.69847000
C	2.86805600	1.53431300	-1.65208000
Si	3.28217200	-1.63877000	0.13670600
C	2.86966700	-3.29984700	1.02188200
C	2.63779500	-4.44771500	0.20463800
C	2.79689500	-3.46260800	2.44275900
C	2.34115600	-5.68413300	0.78889600

C	2.51180300	-4.72962900	2.97405200
C	2.28352000	-5.83329600	2.16711300
H	2.15478100	-6.54434600	0.15454800
H	2.46660500	-4.84890900	4.05207500
H	2.06270800	-6.80189600	2.60877600
C	4.97802300	-1.29368900	-0.71112500
C	6.08635000	-1.02329400	0.14330400
C	5.16687300	-1.13239500	-2.12120100
C	7.28461000	-0.52679900	-0.38376900
C	6.38696700	-0.63253100	-2.59721800
C	7.43311100	-0.30860300	-1.74480800
H	8.11314200	-0.31295300	0.28512700
H	6.52177200	-0.50326300	-3.66631900
H	8.36319800	0.08950100	-2.14235600
C	-2.86961100	-3.29992200	-1.02187000
C	-2.63755000	-4.44774100	-0.20460900
C	-2.79690900	-3.46272000	-2.44274800
C	-2.34081600	-5.68414400	-0.78884900
C	-2.51172100	-4.72972800	-2.97402100
C	-2.28326500	-5.83334700	-2.16706400
H	-2.15429800	-6.54431500	-0.15448500
H	-2.46658100	-4.84903600	-4.05204300
H	-2.06237900	-6.80193700	-2.60871300
C	-4.97800700	-1.29381100	0.71116300
C	-6.08638100	-1.02350800	-0.14323800
C	-5.16682700	-1.13249100	2.12124200
C	-7.28465300	-0.52706900	0.38386000
C	-6.38693700	-0.63268700	2.59728200
C	-7.43312500	-0.30884300	1.74489600
H	-8.11322000	-0.31329800	-0.28501600
H	-6.52171600	-0.50339800	3.66638500
H	-8.36322500	0.08921500	2.14246200
C	3.00629400	-2.36652200	3.49319800
H	3.21378000	-1.42321000	2.98557100
C	4.20173200	-2.67437400	4.42010400
H	4.39433800	-1.82127200	5.08137400
H	3.99780300	-3.54234900	5.05717600
H	5.11449000	-2.88750500	3.85992100
C	1.74613600	-2.15973500	4.36170000
H	1.87100900	-1.27857400	5.00162500
H	0.85141700	-2.01200200	3.75379900
H	1.57291900	-3.02149900	5.01664200
C	2.66136200	-4.39683800	-1.31957000
H	3.18698500	-3.48293100	-1.61456800
C	1.23691600	-4.31576400	-1.89973900
H	0.68386300	-3.46229100	-1.49684900
H	1.27116700	-4.21801600	-2.99161100
H	0.66619700	-5.21984300	-1.66183200
C	3.43166800	-5.57423300	-1.94767800
H	3.50101700	-5.44389300	-3.03189300
H	4.44769300	-5.65347800	-1.54539500
H	2.92673900	-6.53036800	-1.77410300
C	4.17918900	-1.57349200	-3.20538800
H	3.20926100	-1.77085300	-2.74046300
C	3.94958800	-0.53576400	-4.32454800
H	4.82370600	-0.45189500	-4.98018500
H	3.10798500	-0.85077100	-4.95196400
H	3.72778100	0.45728800	-3.93124900
C	4.68704600	-2.88277200	-3.85068800
H	4.92648600	-3.64377900	-3.10475200
H	3.93706300	-3.29223300	-4.53861900
H	5.59957200	-2.69294300	-4.42744200
C	6.07531400	-1.30477700	1.64227400
H	5.08044600	-1.67119200	1.91446000
C	7.07744200	-2.42956700	1.97764000
H	7.07667100	-2.64750500	3.05134000
H	6.83314600	-3.35192100	1.43957400
H	8.09851500	-2.14324700	1.70250600
C	6.34482400	-0.05050500	2.49414800
H	5.63296700	0.74825900	2.26525700

H	6.26095000	-0.28974300	3.56062900
H	7.35412800	0.34111200	2.32370400
C	-3.00647200	-2.36669200	-3.49321600
H	-3.21412200	-1.42340200	-2.98561600
C	-4.20183900	-2.67476600	-4.42014000
H	-3.99774700	-3.54271900	-5.05719200
H	-5.11457100	-2.88804400	-3.85997200
H	-4.39457700	-1.82171000	-5.08142900
C	-1.74633300	-2.15972100	-4.36170200
H	-1.87134000	-1.27859700	-5.00165200
H	-0.85164900	-2.01182500	-3.75379000
H	-1.57296400	-3.02147400	-5.01661800
C	-2.66098400	-4.39683400	1.31959700
H	-3.18678800	-3.48304400	1.61463400
C	-1.23649900	-4.31542300	1.89962700
H	-1.27066800	-4.21768300	2.99150200
H	-0.66558800	-5.21936500	1.66166400
H	-0.68368900	-3.46181700	1.49668400
C	-3.43094900	-5.57440900	1.94778400
H	-3.50015900	-5.44412500	3.03201400
H	-4.44701800	-5.65385400	1.54565100
H	-2.92584400	-6.53043200	1.77409500
C	-4.17909200	-1.57349600	3.20541800
H	-3.20911400	-1.77064200	2.74050400
C	-4.68672900	-2.88292100	3.85059400
H	-3.93667900	-3.29231800	4.53849000
H	-5.59929000	-2.69330200	4.42735900
H	-4.92603400	-3.64389800	3.10458400
C	-3.94972200	-0.53582300	4.32467600
H	-3.10807600	-0.85072200	4.95208900
H	-3.72809800	0.45730700	3.93147400
H	-4.82387600	-0.45218300	4.98029600
C	-6.07539400	-1.30505700	-1.64219500
H	-5.08052200	-1.67143800	-1.91440900
C	-7.07748700	-2.42990800	-1.97746200
H	-6.83311700	-3.35222900	-1.43937400
H	-8.09855800	-2.14362600	-1.70228100
H	-7.07676800	-2.64788700	-3.05115400
C	-6.34500500	-0.05083600	-2.49411200
H	-5.63317700	0.74797300	-2.26528900
H	-6.26116700	-0.29011700	-3.56058700
H	-7.35432000	0.34073900	-2.32363900

3e

C	-0.47229100	2.91732300	-3.47639700
C	-0.20874800	4.27179100	-3.01956000
C	1.06320700	4.61479200	-2.55445000
C	2.12027000	3.61943300	-2.52940000
C	1.86052600	2.30902200	-2.95336600
C	0.53875800	1.95908300	-3.43701500
C	-1.82491300	2.56505000	-3.06958900
C	-2.38683100	3.68353900	-2.35805700
C	-1.39098000	4.74162100	-2.32094600
C	-1.25583700	5.53671400	-1.17900200
C	1.20131600	5.44558300	-1.36965300
C	2.90242600	3.82973000	-1.33797100
C	3.38797300	2.72280000	-0.63088700
C	3.15678100	1.37983100	-1.09234800
C	2.39021100	1.17432800	-2.22775000
C	1.37735000	0.10220800	-2.26936000
C	0.25033400	0.60222800	-3.00359500
C	-1.05459700	0.26584700	-2.60912400
C	-2.10452200	1.26323600	-2.63838000
C	-3.19959000	3.44546800	-1.24113000
C	-3.50573500	2.09542100	-0.82406900
C	-2.98555700	1.01906300	-1.52149600
C	-2.62200200	-0.29181400	-0.88502800
C	-1.27604000	-0.58914500	-1.48463400
C	-0.17588000	-1.03012000	-0.75637100
C	1.16643700	-0.71357400	-1.16341600

C	2.07516100	-0.73455800	0.03310100
C	3.19662000	0.43919700	0.07480000
C	2.34400800	4.96069900	-0.61542200
C	0.93933300	4.58006500	2.64903200
C	-0.35359900	4.23021600	3.04619300
C	0.11082500	0.56290500	2.99896600
C	1.27197400	0.07925900	2.30928300
C	1.13284600	5.42598400	1.48286200
C	0.02801900	5.88432800	0.76053500
C	-1.31217500	5.51954300	1.17376800
C	-1.50081800	4.70863100	2.29651700
C	-2.49447500	3.64848000	2.26823700
C	-1.96594800	2.52106900	2.99003700
C	-2.22075800	1.22602200	2.52569200
C	-1.17310800	0.22656400	2.53971000
C	1.11024000	-0.71707600	1.18112000
C	-0.20814500	-1.04150900	0.71319700
C	-1.34167700	-0.61899300	1.39740400
C	-2.65372600	-0.30865100	0.73281500
C	-3.03977900	0.99844700	1.36113000
C	-3.53161500	2.08282900	0.65533200
C	-3.25075100	3.42627500	1.10900800
C	-3.04957500	4.26897000	-0.05517800
C	-2.10539900	5.29705900	-0.02424500
C	2.31097600	4.95111700	0.77892100
C	0.06292400	5.89468500	-0.69583400
Si	-3.83923700	-1.54098800	-0.11109900
C	3.35940000	2.71439800	0.81871300
C	2.83736100	3.81236300	1.51430800
C	1.72190500	2.27007200	3.05852500
C	1.99864200	3.58641600	2.66410800
C	0.37667000	1.91282900	3.46764200
C	-0.63662500	2.86936600	3.47017700
C	3.11530100	1.36369500	1.25547900
C	2.29155600	1.14556100	2.34707600
Si	3.89138200	-1.33467900	0.08800900
C	4.58692100	-1.89777700	-1.61675600
C	4.16913200	-3.16545200	-2.11526700
C	5.36237600	-1.06103600	-2.48176900
C	4.42377600	-3.52457900	-3.44388700
C	5.59829900	-1.47227200	-3.80105100
C	5.11724500	-2.67693900	-4.29443000
H	4.07636200	-4.48531000	-3.81269600
H	6.18162400	-0.83250500	-4.45537200
H	5.30220100	-2.96088600	-5.32728200
C	4.44586900	-1.92396600	1.83478700
C	5.64205400	-1.36825000	2.37604900
C	3.65711300	-2.76842300	2.67963000
C	5.98004100	-1.58588400	3.71695400
C	4.04607300	-2.96051700	4.01269800
C	5.18220600	-2.36472300	4.54125800
H	6.88536000	-1.13908400	4.11736500
H	3.44214600	-3.59820400	4.65040300
H	5.45095700	-2.52050900	5.58294600
C	-3.16403600	-3.34371600	-0.15794900
C	-2.54430500	-3.92034300	-1.31202500
C	-3.12676400	-4.08241900	1.06003900
C	-1.86021400	-5.13754200	-1.18719900
C	-2.41858700	-5.28835300	1.13480800
C	-1.77186700	-5.81024700	0.02406200
H	-1.39355900	-5.57235400	-2.06520600
H	-2.38677400	-5.83008500	2.07573200
H	-1.22531000	-6.74753200	0.09354100
C	-5.67298300	-0.94761500	-0.09058800
C	-6.30747800	-0.38973500	1.06575100
C	-6.38965100	-0.91667200	-1.32277300
C	-7.56305900	0.21923000	0.93359100
C	-7.63704600	-0.28604300	-1.40155800
C	-8.22055900	0.29408600	-0.28588500
H	-8.03777800	0.64251600	1.81291200

H	-8.15811100	-0.25419300	-2.35390000
H	-9.18669800	0.78656200	-0.36062100
C	3.47045800	-4.20793900	-1.25103300
H	3.41967800	-3.81767000	-0.23014600
C	6.07638900	0.22644000	-2.06231700
H	5.67392000	0.55543100	-1.10143900
C	4.29032700	-5.51279200	-1.18668200
H	3.80214500	-6.24193700	-0.53134600
H	4.38731300	-5.97394200	-2.17554800
H	5.30075100	-5.32946400	-0.80483900
C	2.02735800	-4.48317100	-1.70753400
H	1.53670400	-5.19275700	-1.03119400
H	1.43368500	-3.56486300	-1.72286800
H	2.00637800	-4.91304900	-2.71595100
C	7.58393800	-0.05992200	-1.88170200
H	8.04666200	-0.29710300	-2.84670600
H	8.09756800	0.81840300	-1.47182100
H	7.76345800	-0.90790400	-1.21619000
C	5.90589400	1.39706900	-3.05295500
H	4.86129000	1.55406700	-3.32831000
H	6.28175500	2.32152400	-2.60014600
H	6.47889200	1.23434700	-3.97284300
C	6.63482000	-0.55254200	1.55502600
H	6.26252300	-0.50001600	0.52721100
C	2.43870400	-3.58199000	2.23513800
H	2.12475800	-3.23658200	1.24703900
C	1.21921900	-3.44760700	3.17109400
H	0.98549500	-2.40463500	3.39270400
H	0.34134900	-3.90225300	2.69860600
H	1.38277800	-3.96772000	4.12201900
C	2.81985800	-5.07562300	2.12949500
H	1.99363300	-5.65382400	1.69797800
H	3.71026500	-5.23195100	1.51551600
H	3.03228100	-5.48794700	3.12270100
C	8.00794000	-1.25345200	1.50108500
H	7.92525000	-2.26125000	1.07903900
H	8.70996300	-0.67970500	0.88690800
H	8.44584400	-1.34735500	2.50079400
C	6.77896500	0.89484600	2.06180400
H	7.45028600	1.46309300	1.40696800
H	5.81319600	1.40763000	2.09020500
H	7.20277200	0.92190200	3.07235300
C	-3.87669600	-3.65722500	2.31719200
H	-4.41493600	-2.73255800	2.08996000
C	-2.65856600	-3.37308700	-2.73711900
H	-3.03025100	-2.34698100	-2.68899300
C	-5.77594000	-0.47400000	2.49896300
H	-4.72991600	-0.78802900	2.46882200
C	-5.88836200	-1.57545400	-2.60242500
H	-4.94216800	-2.07397900	-2.37471800
C	-3.67574900	-4.22420500	-3.52931300
H	-4.63041900	-4.31602600	-3.00478200
H	-3.86299900	-3.78384800	-4.51623000
H	-3.28785800	-5.23775400	-3.68430900
C	-1.32857300	-3.32772200	-3.51498100
H	-1.47766200	-2.80489800	-4.46662000
H	-0.54832000	-2.80282800	-2.96128900
H	-0.96158900	-4.33232100	-3.75367200
C	-6.86672100	-2.66740800	-3.08194100
H	-6.47882400	-3.16525200	-3.97687400
H	-7.02802400	-3.42667900	-2.30857400
H	-7.84337900	-2.24299200	-3.33873500
C	-5.61159700	-0.55809500	-3.72534600
H	-4.90272400	0.21065300	-3.40440900
H	-5.19501800	-1.06356000	-4.60486200
H	-6.53287400	-0.05397900	-4.03956900
C	-6.57796200	-1.53316400	3.28730100
H	-6.14003500	-1.68931300	4.28083000
H	-7.61361400	-1.20282500	3.42791600
H	-6.61004800	-2.49447000	2.76801300

C	-5.82433100	0.86433900	3.26610300
H	-5.26703100	0.77245700	4.20521900
H	-5.38776100	1.68297100	2.69077500
H	-6.85139800	1.14318800	3.52745600
C	-4.93717400	-4.70968200	2.70190000
H	-5.50228700	-4.38294100	3.58121200
H	-5.64434300	-4.88119200	1.88276700
H	-4.47333400	-5.67144200	2.94708100
C	-2.94246900	-3.36145400	3.50482500
H	-2.20089500	-2.59826200	3.25127900
H	-3.52120400	-3.00459300	4.36512400
H	-2.40377700	-4.26222300	3.82141100

3trans-1

C	0.00032600	0.69674600	-3.49563300
C	1.17271300	1.42779200	-3.04418600
C	2.29741100	0.72737000	-2.59017300
C	2.29395100	-0.72147600	-2.58545800
C	1.17180500	-1.42480800	-3.04085100
C	-0.00032800	-0.69692800	-3.49561900
C	-1.17181000	1.42464000	-3.04089600
C	-0.72608400	2.59067300	-2.31824500
C	0.72578400	2.59338900	-2.32065500
C	1.42894200	2.99683900	-1.17899100
C	3.05429400	1.17876900	-1.44830600
C	3.04344400	-1.16781900	-1.43932400
C	2.60444900	-2.27740800	-0.73554400
C	1.42791000	-2.99219600	-1.17473000
C	0.72607000	-2.59082100	-2.31816700
C	-0.72579700	-2.59353400	-2.32059000
C	-1.17272000	-1.42796300	-3.04416200
C	-2.29742000	-0.72752200	-2.59017600
C	-2.29396200	0.72132400	-2.58549100
C	-1.42793500	2.99208100	-1.17482500
C	-2.60447400	2.27730400	-0.73562900
C	-3.04347000	1.16770200	-1.43938000
C	-3.74306000	-0.00336800	-0.81344500
C	-3.05430400	-1.17889300	-1.44831000
C	-2.60948000	-2.28551900	-0.74370500
C	-1.42896600	-2.99695700	-1.17892200
C	-0.69745300	-3.43103300	-0.00105900
C	0.69750500	-3.43101200	0.00109300
C	3.74303500	0.00324000	-0.81341800
C	1.17272400	-1.42784700	3.04413800
C	2.29740500	-0.72739600	2.59013400
C	2.29391000	0.72145100	2.58541700
C	1.17174100	1.42475500	3.04079800
C	-0.00037700	0.69684400	3.49555700
C	-1.17179800	-1.42475500	3.04082500
C	-0.72603900	-2.59077800	2.31817900
C	0.72582800	-2.59345500	2.32060000
C	1.42900900	-2.99688800	1.17894400
C	2.60950300	-2.28543000	0.74370700
C	3.05431500	-1.17878100	1.44827500
C	3.04339900	1.16780700	1.43928700
C	2.60438500	2.27739200	0.73550600
C	1.42782600	2.99214900	1.17468400
C	0.72598500	2.59075900	2.31811700
C	-0.72588200	2.59343500	2.32052600
C	-1.17278400	1.42785100	3.04409300
C	-2.29746200	0.72738300	2.59009500
C	-2.29396600	-0.72146400	2.58541900
C	-1.42787100	-2.99220300	1.17475300
C	-2.60442900	-2.27746100	0.73554800
C	-3.04344000	-1.16785400	1.43929600
C	-3.74305200	0.00316800	0.81335200
C	-3.05434800	1.17873800	1.44821800
C	-2.60954100	2.28537200	0.74362500
C	-1.42905300	2.99684200	1.17885300
C	-0.69753900	3.43093200	0.00099400

C	0.69741900	3.43094800	-0.00114600
C	3.74302100	-0.00323300	0.81339200
C	2.60945000	2.28541100	-0.74374200
C	0.00031500	-0.69683000	3.49557300
Si	-5.46690200	-0.00003100	-0.00003000
Si	5.46690300	0.00001500	0.00000300
C	6.30622300	-1.73316600	-0.01829600
C	6.80632300	-2.24888400	1.21315900
C	6.31758000	-2.59736300	-1.15963400
C	7.21705700	-3.58388800	1.30725300
C	6.74920100	-3.92290400	-1.01270300
C	7.17729400	-4.42675300	0.20717100
H	7.57628400	-3.96465300	2.25883800
H	6.75281500	-4.57451700	-1.88056600
H	7.49238500	-5.46347100	0.29393000
C	6.30653800	1.73302100	0.01832800
C	6.80680100	2.24864300	-1.21311400
C	6.31812100	2.59716500	1.15969300
C	7.21801500	3.58349700	-1.30711000
C	6.75029400	3.92254700	1.01286600
C	7.17860500	4.42630200	-0.20696300
H	7.57735900	3.96420800	-2.25867400
H	6.75416700	4.57408900	1.88077900
H	7.49412100	5.46289500	-0.29366200
C	-6.30661300	-1.73298900	0.01834400
C	-6.80699600	-2.24863800	-1.21302900
C	-6.31804800	-2.59711600	1.15972700
C	-7.21811600	-3.58351700	-1.30699400
C	-6.75003800	-3.92256200	1.01292000
C	-7.17843200	-4.42634400	-0.20686900
H	-7.57759300	-3.96423500	-2.25850500
H	-6.75370300	-4.57412600	1.88081800
H	-7.49382400	-5.46297600	-0.29355800
C	-6.30600900	1.73323200	-0.01833900
C	-6.80593300	2.24905100	1.21313200
C	-6.31703900	2.59748900	-1.15963300
C	-7.21611700	3.58420800	1.30733500
C	-6.74807500	3.92321300	-1.01259900
C	-7.17594400	4.42715000	0.20732000
H	-7.57523000	3.96502900	2.25894400
H	-6.75144700	4.57488100	-1.88042100
H	-7.49055900	5.46400400	0.29417200
C	5.98134100	-2.17878600	-2.59320100
H	5.48451600	-1.20598900	-2.56853500
C	7.28492300	-2.03664500	-3.40970800
H	7.76093200	-3.01481100	-3.54443900
H	7.07478200	-1.62793900	-4.40572600
H	8.01020500	-1.38602700	-2.91459400
C	5.03432300	-3.15192200	-3.32651000
H	4.15193200	-3.39307300	-2.73065800
H	4.69788000	-2.70149300	-4.26724700
H	5.53852500	-4.09081400	-3.58186300
C	6.96193600	-1.40650200	2.47413800
H	6.65345100	-0.38399000	2.23732800
C	8.43929200	-1.33954600	2.91289900
H	8.54959300	-0.70494100	3.79852300
H	8.82410900	-2.33249000	3.16971900
H	9.07374600	-0.93335200	2.11726900
C	6.06935100	-1.89629400	3.62995700
H	6.16398100	-1.22867200	4.49470800
H	5.01602700	-1.92960500	3.33669900
H	6.35834800	-2.90214800	3.95602400
C	5.98150900	2.17869800	2.59321800
H	5.48463300	1.20593200	2.56849200
C	7.28482400	2.03653000	3.41014100
H	7.07434000	1.62775100	4.40605600
H	8.01028100	1.38595700	2.91522800
H	7.76079100	3.01468300	3.54511900
C	5.03433100	3.15196900	3.32615700
H	4.15212600	3.39309700	2.73002300

H	4.69758500	2.70167600	4.26685200
H	5.53852200	4.09085800	3.58153200
C	6.96207000	1.40633800	-2.47419300
H	6.65329600	0.38389000	-2.23749000
C	8.43937000	1.33901100	-2.91307600
H	9.07378200	0.93257100	-2.11753300
H	8.54942300	0.70444700	-3.79875900
H	8.82443800	2.33187200	-3.16984900
C	6.06954800	1.89655600	-3.62987700
H	6.16386200	1.22900600	-4.49471700
H	5.01626300	1.93019200	-3.33651100
H	6.35886100	2.90234700	-3.95586000
C	-6.96248700	-1.40626800	-2.47403000
H	-6.65368100	-0.38384400	-2.23728500
C	-5.98154300	-2.17852800	2.59323800
H	-5.48462100	-1.20578900	2.56845700
C	-6.07011500	-1.89639700	-3.62987100
H	-6.16448900	-1.22875800	-4.49463900
H	-5.01679600	-1.93010500	-3.33662000
H	-6.35950200	-2.90214400	-3.95592200
C	-8.43985200	-1.33889900	-2.91264700
H	-8.55007600	-0.70424400	-3.79824300
H	-8.82496400	-2.33173600	-3.16945100
H	-9.07412300	-0.93254000	-2.11694800
C	-7.28496600	-2.03617400	3.40996500
H	-7.76114000	-3.01425000	3.54475800
H	-7.07457700	-1.62752900	4.40595500
H	-8.01021200	-1.38540200	2.91499300
C	-5.03447500	-3.15171400	3.32642900
H	-4.15217800	-3.39290200	2.73045100
H	-4.69787100	-2.70130000	4.26711700
H	-5.53868300	-4.09058000	3.58186000
C	-5.98128600	2.17871000	-2.59325700
H	-5.48458400	1.20584800	-2.56862700
C	-6.96204600	1.40654300	2.47396500
H	-6.65359900	0.38402000	2.23713700
C	-7.28518700	2.03661600	-3.40928800
H	-7.07543800	1.62781800	-4.40535300
H	-8.01034800	1.38610400	-2.91386000
H	-7.76114400	3.01482300	-3.54390400
C	-5.03438100	3.15157900	-3.32706800
H	-4.15175000	3.39274000	-2.73157200
H	-4.69831400	2.70091300	-4.26782600
H	-5.53853900	4.09048400	-3.58245700
C	-8.43959600	1.33976300	2.91210300
H	-9.07378500	0.93373900	2.11617300
H	-8.55037500	0.70510100	3.79762900
H	-8.82433500	2.33276800	3.16882700
C	-6.06988100	1.89606000	3.63021000
H	-6.16491100	1.22830700	4.49481500
H	-5.01643400	1.92933100	3.33737800
H	-6.35891400	2.90187800	3.95635600

3trans-2

C	0.74171900	-3.69834700	-2.09332700
C	-0.25011500	-4.30422400	-1.21951800
C	-1.57383200	-3.86409300	-1.26089900
C	-1.96662800	-2.81328500	-2.18405500
C	-1.01097500	-2.24694700	-3.03998400
C	0.36754900	-2.69222100	-2.98458200
C	1.97732100	-3.58402000	-1.34276400
C	1.76013200	-4.12243900	-0.02094400
C	0.38561400	-4.57400000	0.06148000
C	-0.33690000	-4.40668600	1.24470000
C	-2.32726400	-3.69158000	-0.03251500
C	-2.96285400	-1.99122700	-1.53759100
C	-2.97097500	-0.62107200	-1.74930700
C	-1.95932200	-0.03116800	-2.58623900
C	-1.00633300	-0.81847500	-3.24267500
C	0.37350200	-0.37016600	-3.32548500

C	1.21965800	-1.52411000	-3.16132400
C	2.40381400	-1.40575400	-2.42295000
C	2.78574100	-2.45034900	-1.49711100
C	2.36228000	-3.49723000	1.07910000
C	3.21368100	-2.34181800	0.90098500
C	3.43799400	-1.83632600	-0.36857500
C	3.68643000	-0.38628500	-0.66563500
C	2.82146700	-0.13900600	-1.86878100
C	1.99473800	0.96260500	-2.01161000
C	0.75186100	0.84429900	-2.74206100
C	-0.24420000	1.66544900	-2.07635300
C	-1.57451200	1.23718100	-2.00044700
C	-3.19647500	-2.54552700	-0.18765000
C	-2.00464300	1.46724100	1.64611500
C	-2.81468100	0.35801100	1.82282100
C	-2.39065400	-0.68885700	2.71984600
C	-1.20826700	-0.57700500	3.46177500
C	-0.37542100	0.58486800	3.28979600
C	0.22319500	2.18636500	1.51163600
C	-0.39217300	2.45638700	0.23788900
C	-1.77278100	2.02644700	0.29592100
C	-2.36533900	1.44549900	-0.81072700
C	-3.42678100	0.38507800	-0.73333600
C	-3.68583900	-0.22715800	0.75082100
C	-2.76788000	-1.95931500	2.13885400
C	-1.94392400	-3.07910300	2.31260400
C	-0.70702000	-2.95868200	3.06145300
C	-0.34394700	-1.73662700	3.62858700
C	1.03002900	-1.27853700	3.55701400
C	1.01010800	0.14862000	3.34289400
C	1.95812700	0.72717800	2.49073900
C	1.55652000	1.76446700	1.56124700
C	0.36741900	2.28844900	-0.93091400
C	1.74981200	1.86625500	-0.86595200
C	2.34225400	1.62557200	0.36101200
C	3.42236100	0.61019200	0.59136300
C	2.98549400	-0.06386500	1.86167800
C	2.98673700	-1.43760600	2.04756800
C	1.99504600	-2.05395000	2.89871000
C	1.61194800	-3.32567100	2.30895000
C	0.29351800	-3.77556900	2.39492000
C	-3.43422000	-1.70287900	0.88495100
C	-1.71785600	-3.96948800	1.19839200
C	-0.76507500	1.57694000	2.38273200
Si	5.19187600	0.64822100	-0.11804500
Si	-5.19922100	0.59913000	-0.06321800
C	5.52141000	2.17791500	-1.24100600
C	6.06212600	1.95086800	-2.54039400
C	5.11382100	3.50957800	-0.90890400
C	6.11319100	2.98936900	-3.47779500
C	5.19631700	4.51529400	-1.88188500
C	5.67050300	4.26456600	-3.16154600
H	6.50997000	2.79254200	-4.46942400
H	4.88353100	5.52245100	-1.62567300
H	5.70905200	5.06155300	-3.89974700
C	6.45921000	-0.35832200	0.92587900
C	6.87449500	0.18149300	2.17824500
C	6.88852100	-1.68278500	0.59232200
C	7.61164600	-0.59920900	3.07653000
C	7.63458300	-2.41747800	1.52428100
C	7.98078300	-1.89842800	2.76361000
H	7.90207600	-0.17770300	4.03440300
H	7.95519200	-3.42198000	1.26739300
H	8.54745900	-2.49744100	3.47187700
C	-5.55190800	2.36582700	0.61821400
C	-5.52204400	2.70089100	2.00984200
C	-5.69794800	3.42912400	-0.32020800
C	-5.56911800	4.04855500	2.39262100
C	-5.73095200	4.75904700	0.11568100
C	-5.65280000	5.07538200	1.46324200

H	-5.54359100	4.29680500	3.44879800
H	-5.82277500	5.55677800	-0.61556500
H	-5.67300000	6.11212600	1.78939100
C	-6.44791200	-0.65979600	-0.81515200
C	-7.25315000	-1.42123500	0.08176500
C	-6.50425600	-0.98764100	-2.20767200
C	-8.00768100	-2.50285600	-0.38778200
C	-7.28502300	-2.07412100	-2.62594900
C	-8.01660000	-2.84259300	-1.73187500
H	-8.59927600	-3.08382400	0.31365900
H	-7.32197400	-2.31985300	-3.68236600
H	-8.60109800	-3.68876300	-2.08391600
C	-5.85851100	3.20908400	-1.82010000
H	-5.86693300	2.13075500	-2.00446200
C	-5.53693900	1.69703600	3.16539000
H	-5.31251300	0.70321900	2.77104200
C	-5.84854400	-0.18726400	-3.33578300
H	-5.12450000	0.50574300	-2.90095000
C	-7.38020700	-1.10705300	1.56799600
H	-6.78399000	-0.21369200	1.77612000
C	-5.08599800	-1.05368300	-4.36018600
H	-4.49815700	-0.40949600	-5.02387900
H	-4.40413600	-1.75592800	-3.87679700
H	-5.77164100	-1.62782100	-4.99364300
C	-6.92388300	0.63699600	-4.07764800
H	-7.52215600	1.24246000	-3.39212100
H	-6.45993300	1.30340600	-4.81515300
H	-7.61202000	-0.02639900	-4.61422700
C	-7.21085800	3.76049900	-2.31665100
H	-7.33475700	3.56930500	-3.38775800
H	-8.04992900	3.29783600	-1.78518300
H	-7.27924600	4.84333900	-2.16578700
C	-4.69335600	3.80214800	-2.63450700
H	-3.72957100	3.40652100	-2.30115500
H	-4.81220800	3.56618000	-3.69876600
H	-4.65835600	4.89370600	-2.53997000
C	-4.49748000	1.99157900	4.26742800
H	-4.44389700	1.14437000	4.96056400
H	-3.49985500	2.15805200	3.85660200
H	-4.77322900	2.87289100	4.85751300
C	-6.94445600	1.65990400	3.80074800
H	-7.00766700	0.86539600	4.55438400
H	-7.16462400	2.61077800	4.29967700
H	-7.72662500	1.49336900	3.05582500
C	-6.83743900	-2.23724200	2.46275700
H	-5.79669100	-2.47381900	2.22307800
H	-6.88903400	-1.94636900	3.51878000
H	-7.42543300	-3.15468600	2.34374500
C	-8.83937200	-0.76893900	1.93683200
H	-8.91832300	-0.51032500	2.99799100
H	-9.21444600	0.07549000	1.34792200
H	-9.50376800	-1.62080900	1.75566300
C	4.66766100	3.98193000	0.47720300
H	4.45959400	3.10667500	1.09715300
C	6.64000100	0.61260200	-2.98649300
H	6.58283400	-0.07874200	-2.14049600
C	8.13202100	0.75122100	-3.35274600
H	8.55002200	-0.21946500	-3.63946300
H	8.27313400	1.43280300	-4.19860200
H	8.71470200	1.14180800	-2.51101000
C	5.84761800	-0.01899400	-4.14656300
H	6.25576100	-1.00595600	-4.39507000
H	4.79075100	-0.13980100	-3.89132500
H	5.90545600	0.59946800	-5.04976100
C	5.80664300	4.78698600	1.14116800
H	5.97404600	5.72781400	0.60416800
H	5.55050200	5.03594500	2.17831000
H	6.75173400	4.23819900	1.14032500
C	3.38688700	4.84289400	0.46567500
H	2.58446800	4.37690000	-0.10937300

H	3.03082400	4.98886700	1.49185300
H	3.57287700	5.83771500	0.04533600
C	6.58863700	1.61522300	2.61020700
H	6.05807500	2.11452200	1.79398400
C	6.68667700	-2.36690000	-0.76216700
H	5.94837500	-1.80126600	-1.33550300
C	5.68569900	1.69128300	3.85575900
H	5.45362900	2.73577000	4.09592300
H	4.74326500	1.15785900	3.70102700
H	6.17902800	1.25283200	4.73095700
C	7.90072900	2.39442700	2.83563800
H	8.52222100	2.39867100	1.93324800
H	7.68943500	3.43257300	3.11279800
H	8.49285500	1.95218700	3.64418200
C	6.16826700	-3.81709300	-0.66288600
H	5.29943100	-3.89840400	-0.00698100
H	5.87936200	-4.17500600	-1.65763000
H	6.94126100	-4.49760200	-0.28828300
C	8.01718500	-2.36297000	-1.54702200
H	7.86718800	-2.74442100	-2.56448800
H	8.45177600	-1.36237900	-1.61306200
H	8.75404000	-3.00797500	-1.05453800

3trans-3

C	-1.70335400	-4.80556900	-0.04952200
C	-0.61227900	-5.12955800	-0.95013900
C	0.69729500	-5.18445800	-0.46671100
C	0.96708500	-4.91512200	0.93806700
C	-0.08598200	-4.60402200	1.80115600
C	-1.44377800	-4.54974100	1.29742800
C	-2.65081700	-3.97807600	-0.77997500
C	-2.15154700	-3.79057400	-2.11884900
C	-0.88973600	-4.49789100	-2.23173100
C	0.15666700	-3.94851100	-2.97653100
C	1.78065200	-4.61231900	-1.24495000
C	2.21498500	-4.17999800	1.02158300
C	2.35759100	-3.14361700	1.95577500
C	1.26398200	-2.82332300	2.84947200
C	0.06720900	-3.53854100	2.78201600
C	-1.20158800	-2.84155900	2.89234700
C	-2.12443300	-3.45453000	1.97507400
C	-3.02714400	-2.65182500	1.26636700
C	-3.29518600	-2.91707300	-0.13165400
C	-2.31557200	-2.54464800	-2.74110900
C	-3.01256400	-1.46672700	-2.06968100
C	-3.51762200	-1.65454000	-0.79429700
C	-3.61489300	-0.56491700	0.23649300
C	-3.07973600	-1.22715800	1.47265400
C	-2.18213100	-0.63459300	2.34571000
C	-1.22371600	-1.44957000	3.06065400
C	0.02154400	-0.71383000	3.15179500
C	1.24751900	-1.38250600	3.04390400
C	2.71349600	-3.99303900	-0.31714900
C	2.86097700	0.79657800	0.38295000
C	3.62180300	-0.41803700	-0.37924800
C	3.10820500	-0.73162000	-1.75701800
C	2.19381200	0.05524800	-2.43718500
C	1.49897800	1.15659500	-1.74042700
C	0.64543400	1.70294900	0.47860500
C	0.91247500	1.09220800	1.75344800
C	2.18659800	0.42177600	1.67355400
C	2.34251600	-0.80617600	2.29458300
C	3.07111000	-2.16259500	-1.93622500
C	2.17753500	-2.76131900	-2.83402600
C	1.24133700	-1.94011700	-3.55423200
C	1.24244000	-0.55410500	-3.34238000
C	-0.01642200	0.15452500	-3.22612500
C	0.14227500	1.19479900	-2.24257300
C	-0.93013900	1.49882700	-1.38977200
C	-0.67261900	1.75969500	0.00090100

C	-0.15526200	0.55345000	2.48864800
C	-1.51281600	0.63293400	1.99040800
C	-1.77412900	1.24478100	0.77795200
C	-2.87322100	0.82421400	-0.15596000
C	-2.18902300	0.80795500	-1.49233900
C	-2.33152200	-0.20667900	-2.42430800
C	-1.23122300	-0.53849900	-3.30272000
C	-1.22620800	-1.97685100	-3.50904400
C	-0.01649000	-2.66205200	-3.63549300
C	3.33594600	-2.78566700	-0.65633900
C	1.51483100	-4.00840400	-2.47430900
C	1.74854400	1.42088200	-0.40582400
Si	-4.74864000	0.96663400	0.16508400
C	3.03009500	-1.91168200	1.59969500
C	3.52956300	-1.74285800	0.31940300
Si	4.73237400	1.05244700	0.11184300
C	5.12897700	2.23616200	-1.35473600
C	6.04491400	1.78474400	-2.34952900
C	4.44446000	3.47191300	-1.58673300
C	6.19999000	2.49669600	-3.54492700
C	4.64481500	4.15101500	-2.79648400
C	5.49558700	3.66736300	-3.78014100
H	6.88732300	2.12638000	-4.29986600
H	4.12181900	5.08638600	-2.96805100
H	5.61946900	4.20825900	-4.71484300
C	5.78704900	0.71148500	1.68660500
C	5.73661500	1.66439900	2.74575800
C	6.49283600	-0.51203700	1.91997900
C	6.29585000	1.36630200	3.99396900
C	7.04391700	-0.75569400	3.18564600
C	6.93521000	0.15784800	4.22427400
H	6.23019300	2.09677100	4.79500000
H	7.57554900	-1.68598900	3.35794300
H	7.35991700	-0.06531300	5.19966900
C	-5.78750700	1.07817800	-1.45235900
C	-6.88122700	0.17693800	-1.60695200
C	-5.43428900	1.90034200	-2.56985900
C	-7.52832000	0.05368100	-2.84215900
C	-6.12270300	1.74458700	-3.78094200
C	-7.14646700	0.82034000	-3.93238300
H	-8.34638100	-0.65298500	-2.94660900
H	-5.84938200	2.36861300	-4.62575200
H	-7.65197800	0.71135600	-4.88849400
C	-5.17523900	1.67422200	1.90462700
C	-5.54848600	0.86256400	3.02331700
C	-4.95887700	3.06474100	2.13256000
C	-5.62119300	1.44216300	4.29765200
C	-5.03533100	3.59063000	3.42762500
C	-5.35026800	2.78596200	4.51188600
H	-5.90202300	0.82180600	5.14268100
H	-4.84802000	4.64885900	3.58473300
H	-5.40055800	3.20412100	5.51399200
C	3.55565300	4.19812000	-0.57386400
H	3.31725200	3.51116500	0.24146600
C	4.32056300	5.40355900	0.01590500
H	4.48890800	6.16461100	-0.75483400
H	3.74413500	5.86940300	0.82462600
H	5.29859900	5.11579900	0.40961000
C	2.21440200	4.69194800	-1.15598200
H	1.69109600	3.90545500	-1.70312500
H	1.56322400	5.03447800	-0.34362200
H	2.35667500	5.54096200	-1.83420900
C	6.92461500	0.55108200	-2.18209500
H	6.73548400	0.13389500	-1.18850400
C	8.41925700	0.92924900	-2.23300900
H	9.04575000	0.04471000	-2.07736300
H	8.68929400	1.35658300	-3.20481800
H	8.67030200	1.66842800	-1.46414200
C	6.60346400	-0.55107700	-3.20905100
H	7.21195500	-1.44247500	-3.01529800

H	5.54937000	-0.84094900	-3.17022000
H	6.82172000	-0.21769700	-4.23022800
C	6.79588500	-1.57775700	0.86374500
H	6.17518200	-1.38853900	-0.01525900
C	8.27837500	-1.48033400	0.44078400
H	8.48702400	-2.15792400	-0.39620000
H	8.55413900	-0.46587700	0.14195800
H	8.93321100	-1.76610100	1.27208000
C	6.50009200	-3.02066700	1.32428000
H	5.50315500	-3.11647900	1.75857500
H	6.56642800	-3.70243900	0.46882600
H	7.22854300	-3.36491500	2.06709800
C	5.12104200	3.05118500	2.59816800
H	4.77647100	3.15934000	1.56541300
C	3.89801200	3.25835500	3.51127800
H	3.44906200	4.24241200	3.33057000
H	3.13351600	2.49574700	3.33657900
H	4.18062100	3.21352000	4.56944200
C	6.17633700	4.15056400	2.83734800
H	7.02233800	4.04828000	2.14859800
H	5.73666700	5.14367800	2.69714000
H	6.57237500	4.10733400	3.85775900
C	-4.67023800	4.06258200	1.01669000
H	-4.69527600	3.52231300	0.06563400
C	-5.76706500	5.14495500	0.94738300
H	-5.80493100	5.73381500	1.87029600
H	-5.57231300	5.83851100	0.12260400
H	-6.75708900	4.70042600	0.79665500
C	-3.27529100	4.70388800	1.14050200
H	-2.48708000	3.94586000	1.16950400
H	-3.08299600	5.36708400	0.28860400
H	-3.19525100	5.30596300	2.05307600
C	-5.98009700	-0.60447800	2.95162000
H	-5.71224400	-1.00261200	1.97002800
C	-5.30823100	-1.50950600	4.00577100
H	-5.52418900	-2.56033800	3.78183000
H	-4.22420300	-1.38223000	4.02538700
H	-5.69182300	-1.31001300	5.01274500
C	-7.51344700	-0.70145800	3.11227200
H	-7.85486400	-1.73089900	2.94839000
H	-7.81162200	-0.40888500	4.12569500
H	-8.04161500	-0.04647900	2.41482500
C	-7.43709600	-0.67016600	-0.46801300
H	-6.87391700	-0.42837200	0.43838500
C	-7.27435600	-2.18135100	-0.71763700
H	-6.22895300	-2.44773900	-0.89874400
H	-7.62884200	-2.75194500	0.14907200
H	-7.85787300	-2.50493100	-1.58734500
C	-8.91205700	-0.31628500	-0.18743900
H	-9.29549800	-0.90276100	0.65408700
H	-9.02867100	0.74654500	0.05207200
H	-9.54556800	-0.53246400	-1.05452300
C	-4.40689400	3.03503500	-2.54810000
H	-3.80709100	2.94945900	-1.63893500
C	-3.42640700	3.01729400	-3.73969700
H	-2.61900400	3.73741100	-3.56443200
H	-2.97777600	2.03305600	-3.88685100
H	-3.92024300	3.30876700	-4.67357800
C	-5.13848400	4.39524600	-2.52075600
H	-5.87728900	4.44781800	-1.71712600
H	-4.42326300	5.21654000	-2.38933100
H	-5.66939400	4.56360400	-3.46487000

3trans-4

C	1.47933000	3.71026100	2.94631100
C	0.76159000	4.78430700	2.27967000
C	-0.63532100	4.79905400	2.28832700
C	-1.36746800	3.74044800	2.96387600
C	-0.67635700	2.71155200	3.60433200
C	0.77520300	2.69595800	3.59572800

C	2.64073200	3.38489400	2.13647900
C	2.64817500	4.25011600	0.98228900
C	1.48508300	5.11521900	1.06304800
C	0.77985400	5.44654700	-0.09637000
C	-1.36674200	5.14593900	1.08105600
C	-2.54695600	3.44107900	2.16958600
C	-2.98015300	2.11620100	2.03463900
C	-2.25681700	1.04942500	2.69038400
C	-1.13235800	1.33655900	3.47142500
C	0.02459100	0.48084700	3.38528600
C	1.20083300	1.31134100	3.45862700
C	2.31040800	0.99897700	2.66564400
C	3.04171300	2.05093100	1.99588100
C	3.05866700	3.73640800	-0.25379500
C	3.50794900	2.36587500	-0.37753200
C	3.51585100	1.53864300	0.73134700
C	3.27333400	0.05837100	0.67673800
C	2.31830600	-0.16691500	1.81287600
C	1.17680600	-0.94466600	1.72282600
C	0.00813300	-0.61557800	2.50930900
C	-1.17460100	-0.91627000	1.73290400
C	-2.29693400	-0.11278200	1.83535500
C	-2.54987000	4.30686900	1.01570900
C	-2.28560700	2.98086200	-2.30363500
C	-1.16732300	2.68928500	-3.09875300
C	-0.72194400	-0.75614700	-1.89331000
C	-1.45045100	-1.13535800	-0.72412700
C	-2.78686100	-0.44391800	-0.72521800
C	-3.26713800	0.12806800	0.71670500
C	-2.26309200	4.13710400	-1.42806200
C	-1.13075400	4.95263400	-1.37007900
C	0.03420800	4.63610000	-2.17622200
C	0.01783700	3.52688400	-3.02466700
C	1.18483000	2.66559100	-3.11576700
C	0.72272200	1.30766800	-3.23730000
C	1.41195000	0.28608500	-2.57881800
C	0.68310700	-0.76878200	-1.90219200
C	-0.73343800	-1.44012000	0.42683100
C	0.70884000	-1.45197800	0.41903100
C	1.41627900	-1.15621000	-0.74028900
C	2.76714100	-0.49837600	-0.76035500
C	2.61159800	0.55304600	-1.82299600
C	3.04451800	1.86206800	-1.68904600
C	2.31967100	2.93345000	-2.33636400
C	2.32948400	4.08846800	-1.45841400
C	1.21536400	4.92789300	-1.38519200
C	-2.98664100	3.80188400	-0.21497700
C	-0.66864100	5.46205800	-0.08714200
Si	4.40220300	-1.24678400	-0.13314000
C	-3.48318200	1.61368800	0.77658400
C	-3.46733200	2.44131700	-0.33166000
C	-2.61506400	0.60852300	-1.78237400
C	-3.02136500	1.92539300	-1.64381300
C	-1.43653000	0.31536900	-2.55841500
C	-0.73499500	1.32269200	-3.22618000
Si	-4.43269400	-1.15419900	-0.08030000
C	4.29085100	-3.02854400	0.59664400
C	4.67728500	-3.29456300	1.94833000
C	3.86276800	-4.13740600	-0.20061600
C	4.74173300	-4.61604900	2.40955900
C	3.94818600	-5.43851000	0.31305300
C	4.40798600	-5.68858100	1.59691200
H	5.04658900	-4.80340100	3.43465400
H	3.63137500	-6.27201900	-0.30640500
H	4.47517100	-6.70704400	1.97094100
C	5.92391300	-0.42539000	-0.99231700
C	6.79954400	0.44414600	-0.27372400
C	6.05981900	-0.47598400	-2.41215800
C	7.65675500	1.30419300	-0.97244300
C	6.93241800	0.40429500	-3.06451200

C	7.70654600	1.31331500	-2.35871400
H	8.30669400	1.97126500	-0.41445300
H	7.01257300	0.36556800	-4.14672200
H	8.36650400	2.00011900	-2.88242200
C	-4.26143100	-2.91445100	0.68285200
C	-4.05824300	-4.01078900	-0.20558200
C	-4.17402600	-3.16768000	2.08916600
C	-3.70838900	-5.27128400	0.29284800
C	-3.83086600	-4.45158100	2.53531500
C	-3.57954000	-5.49427100	1.65514800
H	-3.53842300	-6.08969900	-0.40062900
H	-3.76353500	-4.63742500	3.60244700
H	-3.30141100	-6.47581600	2.03045700
C	-5.97858800	-0.36362500	-0.91284200
C	-7.00466200	0.15057400	-0.06733600
C	-6.08556000	-0.11969300	-2.31954300
C	-8.03381100	0.93622000	-0.59966000
C	-7.14243700	0.66493100	-2.80154600
C	-8.09999600	1.20886100	-1.95742000
H	-8.79558500	1.33629500	0.06304100
H	-7.21707800	0.84950500	-3.86839200
H	-8.89980300	1.82610900	-2.35852100
C	-4.23258300	-3.90941000	-1.71663500
H	-4.55631000	-2.89113000	-1.95172300
C	-4.51699100	-2.16650900	3.19543200
H	-4.58944800	-1.16845600	2.75681900
C	-5.18410700	-0.72843500	-3.39673200
H	-4.29978200	-1.15382700	-2.91632300
C	-7.08014000	-0.13482900	1.42823400
H	-6.24899300	-0.79725700	1.68788800
C	-5.93842600	-1.86673300	-4.11894700
H	-6.77660200	-1.46298500	-4.69855800
H	-5.27271000	-2.39069800	-4.81581000
H	-6.34881600	-2.59701300	-3.41702900
C	-4.68471600	0.28637000	-4.44667900
H	-4.25980600	1.17959600	-3.98479800
H	-3.91048700	-0.17674600	-5.06894600
H	-5.49079300	0.60218600	-5.11858700
C	-8.37996300	-0.88751900	1.77981300
H	-8.41375800	-1.11921700	2.84959400
H	-9.26479600	-0.28570200	1.54566000
H	-8.46023200	-1.82687300	1.22146700
C	-6.93532700	1.13749400	2.28416200
H	-6.93191500	0.88202600	3.35053800
H	-6.00755800	1.67061000	2.05660600
H	-7.76902200	1.82823800	2.11268800
C	-5.88669000	-2.52528000	3.81302700
H	-6.20308900	-1.75426800	4.52621600
H	-6.66411000	-2.63323800	3.05255500
H	-5.82527800	-3.47583300	4.35547500
C	-3.46444100	-2.09095900	4.32153600
H	-2.45273900	-1.96831700	3.93014500
H	-3.68291200	-1.23869500	4.97500400
H	-3.47916200	-2.98935900	4.94893300
C	-2.91968100	-4.15843200	-2.48269800
H	-3.07231600	-4.01356500	-3.55894800
H	-2.12948500	-3.47734900	-2.15351500
H	-2.56254300	-5.18424000	-2.33452700
C	-5.34369400	-4.85934200	-2.20889000
H	-6.29811000	-4.64721900	-1.71428100
H	-5.48651100	-4.75759000	-3.28983300
H	-5.09125800	-5.90611700	-2.00757700
C	3.22982700	-4.03299500	-1.58938200
H	3.20166700	-2.98154900	-1.88380300
C	4.96893400	-2.22598900	3.00203800
H	4.88215000	-1.24296600	2.53373300
C	6.96764800	0.42862000	1.24254200
H	6.17822700	-0.19487000	1.66795000
C	5.38656600	-1.52178200	-3.29455800
H	4.68830800	-2.09003000	-2.67598800

C	6.45057600	-2.51086700	-3.81933700
H	5.98631600	-3.29124300	-4.43199600
H	7.18936500	-1.99379600	-4.44191700
H	6.99077500	-2.99256800	-2.99679300
C	4.58452600	-0.93214300	-4.46955600
H	4.06097600	-1.73276500	-5.00549100
H	3.84108100	-0.20771600	-4.12856700
H	5.23761300	-0.43089300	-5.19303200
C	8.32252600	-0.22458000	1.59508800
H	9.15290300	0.37118100	1.19927600
H	8.44893400	-0.29504800	2.68095700
H	8.40727600	-1.23094200	1.17100600
C	6.86707900	1.81473500	1.90705100
H	5.94409400	2.33014600	1.63289700
H	6.88774200	1.70828300	2.99805600
H	7.70966000	2.45828700	1.62962200
C	6.38570000	-2.35606700	3.59291400
H	7.15113200	-2.39183200	2.81363200
H	6.60446800	-1.50749500	4.25180200
H	6.48199800	-3.26765900	4.19344700
C	3.92234000	-2.26372700	4.13688200
H	4.08229400	-1.42540600	4.82517600
H	2.90448300	-2.19369200	3.74549300
H	3.99998900	-3.19026400	4.71732200
C	4.02483200	-4.81392500	-2.65399700
H	3.62638800	-4.60973800	-3.65497900
H	5.08565400	-4.55175100	-2.64610100
H	3.95130800	-5.89476800	-2.48859000
C	1.76491300	-4.52172900	-1.57186200
H	1.17796000	-4.00442700	-0.80922000
H	1.29628600	-4.33879900	-2.54615000
H	1.70846400	-5.59804100	-1.37232600
